# Emergency department personnel patient care-related COVID-19 risk

**DOI:** 10.1371/journal.pone.0271597

**Published:** 2022-07-22

**Authors:** Nicholas M. Mohr, Anusha Krishnadasan, Karisa K. Harland, Patrick Ten Eyck, William R. Mower, Walter A. Schrading, Juan Carlos C. Montoy, L. Clifford McDonald, Preeta K. Kutty, Elisabeth Hesse, Scott Santibanez, David N. Weissman, Patricia Slev, David A. Talan, for the Project COVERED Emergency Department Network

**Affiliations:** 1 Department of Emergency Medicine, University of Iowa Carver College of Medicine, Iowa City, Iowa, United States of America; 2 Olive View-UCLA Education and Research Institute, Los Angeles, California, United States of America; 3 Institute for Clinical and Translational Science, University of Iowa Carver College of Medicine, Iowa City, Iowa, United States of America; 4 Department of Emergency Medicine, Ronald Reagan-UCLA Medical Center, Los Angeles, California, United States of America; 5 Department of Emergency Medicine, University of Alabama and Birmingham, Birmingham, Alabama, United States of America; 6 Department of Emergency Medicine, University of California-San Francisco, San Francisco, California, United States of America; 7 Division of Healthcare Quality Promotion, Centers for Disease Control and Prevention, Atlanta, Georgia, United States of America; 8 Division of Preparedness and Emerging Infections, Centers for Disease Control and Prevention, Atlanta, Georgia, United States of America; 9 Respiratory Health Division, National Institute for Occupational Safety and Health, Centers for Disease Control and Prevention, Morgantown, West Virginia, United States of America; 10 ARUP Laboratories, Salt Lake City, Utah, United States of America; Stanford University School of Medicine, UNITED STATES

## Abstract

**Objectives:**

Emergency department (ED) health care personnel (HCP) are at risk of exposure to SARS-CoV-2. The objective of this study was to determine the attributable risk of SARS-CoV-2 infection from providing ED care, describe personal protective equipment use, and identify modifiable ED risk factors. We hypothesized that providing ED patient care increases the probability of acquiring SARS-CoV-2 infection.

**Methods:**

We conducted a multicenter prospective cohort study of 1,673 ED physicians, advanced practice providers (APPs), nurses, and nonclinical staff at 20 U.S. centers over 20 weeks (May to December 2020; before vaccine availability) to detect a four-percentage point increased SARS-CoV-2 incidence among HCP related to direct patient care. Participants provided monthly nasal and serology specimens and weekly exposure and procedure information. We used multivariable regression and recursive partitioning to identify risk factors.

**Results:**

Over 29,825 person-weeks, 75 participants (4.5%) acquired SARS-CoV-2 infection (31 were asymptomatic). Physicians/APPs (aOR 1.07; 95% CI 0.56–2.03) did not have higher risk of becoming infected compared to nonclinical staff, but nurses had a marginally increased risk (aOR 1.91; 95% CI 0.99–3.68). Over 99% of participants used CDC-recommended personal protective equipment (PPE), but PPE lapses occurred in 22.1% of person-weeks and 32.1% of SARS-CoV-2-infected patient intubations. The following factors were associated with infection: household SARS-CoV-2 exposure; hospital and community SARS-CoV-2 burden; community exposure; and mask non-use in public. SARS-CoV-2 intubation was not associated with infection (attributable risk fraction 13.8%; 95% CI -2.0–38.2%), and nor were PPE lapses.

**Conclusions:**

Among unvaccinated U.S. ED HCP during the height of the pandemic, the risk of SARS-CoV-2 infection was similar in nonclinical staff and HCP engaged in direct patient care. Many identified risk factors were related to community exposures.

## Introduction

As of May 2021, over 115,000 health care personnel (HCP) died of SARS-CoV-2 infection worldwide [1, 2]. SARS-CoV-2 spreads primarily through close personal contact via droplets and aerosol transmission, and front-line HCP, such as emergency department (ED) personnel, are at particular risk [3]. According to serology screening prior to vaccine availability, the prevalence of SARS-CoV-2 infection among U.S. hospital-based HCP ranged from 1.1% to 36%, with 38% of ED HCP not recognizing their infection and continuing to work [4, 5].

ED HCP may have additional risk due to unknown patient infection status, overcrowded facilities lacking adequate ventilation, personal protective equipment (PPE) shortages, unexpected critically ill patient arrivals, and performance of life-saving aerosolizing procedures, such as endotracheal intubation [6, 7]. Risk of acquiring infection after intubating a SARS-CoV-2-infected patient has been estimated as high as 3% [8, 9]. Even after availability of SARS-CoV-2 vaccination, the Omicron variant has led to widespread infection, primarily from immune evasion, and SARS-CoV-2 infections in vaccinated HCP have been reported [10, 11]. The COVID-19 pandemic threatens the adequacy of the HCP workforce through both patient-to-HCP and HCP-to-HCP transmission, so optimizing infection mitigation strategies are critical.

Multiple cross-sectional studies have evaluated risk factors for ED HCP SARS-CoV-2 acquisition, with inconsistent findings for the risk attributable to occupational exposures [12–14]. Therefore, we conducted a 20-week prospective surveillance study of U.S. ED HCP during the height of the COVID-19 pandemic, prior to vaccine availability, and prior to emergence of the Delta variant to 1) determine the attributable risk of infection from providing ED care, especially related to high-risk procedures, 2) describe PPE use, and 3) identify modifiable infection risk factors. We hypothesized that providing ED patient care increases the probability of acquiring SARS-CoV-2 infection and that performing aerosol-generating procedures is a specific risk factor.

## Materials and methods

### Study design, setting, and selection of participants

The COVID-19 Evaluation of Risks in Emergency Departments (Project COVERED) was a multicenter prospective cohort study with 20 weeks of continuous observation and serial SARS-CoV-2 serology and reverse transcription polymerase chain reaction (RT-PCR) testing at 20 U.S. EDs at geographically diverse university-affiliated medical centers (protocol available in S1 File). Some sites started observation on May 13, with all sites completing observation by December 9, 2020. The U.S. Centers for Disease Control and Prevention (CDC) reviewed this activity, which was conducted consistent with applicable federal law and CDC policy [15]. All institutional review boards concurred with the activity being public health surveillance, and HCP provided informed consent prior to their participation. This manuscript is reported in accordance with the Strengthening the Reporting of Observational Studies in Epidemiology (STROBE) statement [16].

Through local advertising, we recruited approximately 80 ED HCP volunteers who had not been previously diagnosed with SARS-CoV-2 infection at each site. Investigators attempted to recruit 20 HCP from each of four cohorts: 1) physicians/advanced practice providers (APPs) likely to intubate patients with SARS-CoV-2 infection; 2) physicians/APPs unlikely to intubate SARS-CoV-2 patients; 3) nurses; and 4) non-clinical staff (including clerks, case managers, and others without routine clinical contact). At enrollment, participants had SARS-CoV-2 serology and RT-PCR on nasal swabs performed and anyone with a positive test result was excluded and replaced with a test-negative HCP. We replaced participants who left a risk group (e.g., job change) or were nonadherent with project procedures by another HCP within the same cohort.

### Measurements

During the 20-week surveillance period, each participant completed a detailed baseline survey that ascertained risk factors, routine PPE practices, and non-occupational exposures. Participants completed weekly surveys to track SARS-CoV-2 exposures, behavior changes, and non-occupational exposures. Physicians/APPs completed a detailed report for every intubation and cardiopulmonary resuscitation (CPR) in which they participated (defined as being within three feet of the intubation or personally performing CPR) [17]. Guideline adherence was defined as using PPE recommended by CDC during the surveillance period (surgical or N95 facemask for all patient encounters; eye protection, gown, and gloves additionally for all SARS-CoV-2 patient encounters; and N95 respirator additionally for all SARS-CoV-2 aerosol-generating procedures). During the first four weeks, all sites verified that ≥95% of intubation and CPR events were reported, and monthly auditing ensured ≥95% capture rates.

Participants provided a blood sample and proctored self-administered nasal swab at enrollment, week two, week four, then every four weeks through week 20, which we shipped to a central laboratory (ARUP Laboratories, Salt Lake City, UT). Nasal swabs were analyzed by SARS-CoV-2 RT-PCR. Anti-SARS-CoV-2 IgG (nucleocapsid phosphoprotein) was measured using the Architect i2000 (Abbott Laboratories, Chicago, Illinois), with positive serology results confirmed by orthogonal testing using a spike glycoprotein ELISA assay (EUROIMMUN, Lubeck, Germany). Positive RT-PCR results were confirmed with serology at three and six weeks after the positive test. Participants reported RT-PCR tests performed outside the study for clinical indications. Results were reported back to participants within 1 week of test arrival at the central laboratory.

Site coordinators recorded SARS-CoV-2 test results for intubated and cardiac arrest patients and weekly SARS-CoV-2 infection ED and hospital case counts. We collected weekly cumulative community SARS-CoV-2 infection incidence from public health reports collated from local health department data for each facility’s health service area [18].

### Outcomes

Our primary outcome was new SARS-CoV-2 infection (symptomatic and asymptomatic) defined as a positive nasal RT-PCR (on either a study test or confirmed from outside) or positive nucleocapsid and spike IgG assay serology.

### Analysis

We conducted our analyses at the participant time epoch level. We defined epochs using RT-PCR and serology dates and represent the window during which a participant’s SARS-CoV-2 exposure was most likely to affect a particular test result. We defined these time windows a priori as the median time period from SARS-CoV-2 infection symptom onset to a positive test, lagged by a median four day incubation period [19–21]. For each RT-PCR test, the attributable epoch started three days before the prior RT-PCR test and extended to four days before the current test to make epochs mutually exclusive. We defined serology epochs from 11 days before the prior serology to 12 days before the current test (S1 Fig). We defined a SARS-CoV-2—positive epoch by a positive RT-PCR test, unless the serology was positive before an RT-PCR, in which case serology defined epoch dates.

We conducted analysis of HCP SARS-CoV-2 infection risk with 15 a priori-defined risk factors using regression modeling and recursive partitioning to explore which factors were independently associated with infection. We selected potential risk factors based on extant literature and study team consensus and included job cohort, infection risks related to occupational and non-occupational exposures, and PPE use (S1 Table).

We examined risk by epoch-specific exposures and adjusted for occupational and non-occupational community factors. We used generalized linear mixed modeling (GLMM) and recursive partitioning to assess risk of SARS-CoV-2 infection for individual, facility, and community fixed-effect factors within each ED HCP epoch (S2 File). We calculated attributable risk fraction (AF) using adjusted odds ratio estimates from regression, where p_e_ is exposure prevalence), and we interpreted AF as the fraction of total SARS-CoV-2 infections attributable to a particular risk factor [22, 23].

Missing or delayed RT-PCR or serology testing resulted in some epochs spanning greater than four weeks. If survey data were missing, we carried exposure variables into the subsequent week. We assumed complete reporting of intubation data with multiple verifications, so we did not adjust for missing reports. We classified any intubated or cardiac arrest patient with no SARS-CoV-2 test performed as not having SARS-CoV-2 infection, and we conducted an a priori-defined sensitivity analysis excluding intubations and cardiac arrest cases in which no SARS-CoV-2 test was performed.

We estimated the sample size to detect a four-percentage point increased risk for a participant to develop a new infection between clinical and non-clinical cohorts (e.g., clinical ED HCP participating in aerosol-generating procedures versus nonclinical staff), assuming a 1% non-occupational SARS-CoV-2 acquisition risk over 20 weeks. Assuming 25% loss-to-follow-up and within-site clustering, we estimated we needed 1600 participants to have 90% power to detect this attributable risk (0.2% additional risk per person-week).

We performed all analyses using SAS v.9.4 software (SAS Institute, Cary, NC). We summarized categorical results using summary statistics (e.g., medians, ranges) and used point estimates and 95% CIs to describe infection incidence. We defined statistical significance as p<0.05 for comparison of SARS-CoV-2 infection incidence between ED HCP groups and the non-clinical cohort. We used differences and 95% CIs to describe secondary incidence comparisons, including by epoch and with risk factor adjustment.

## Results

**Fig 1:**
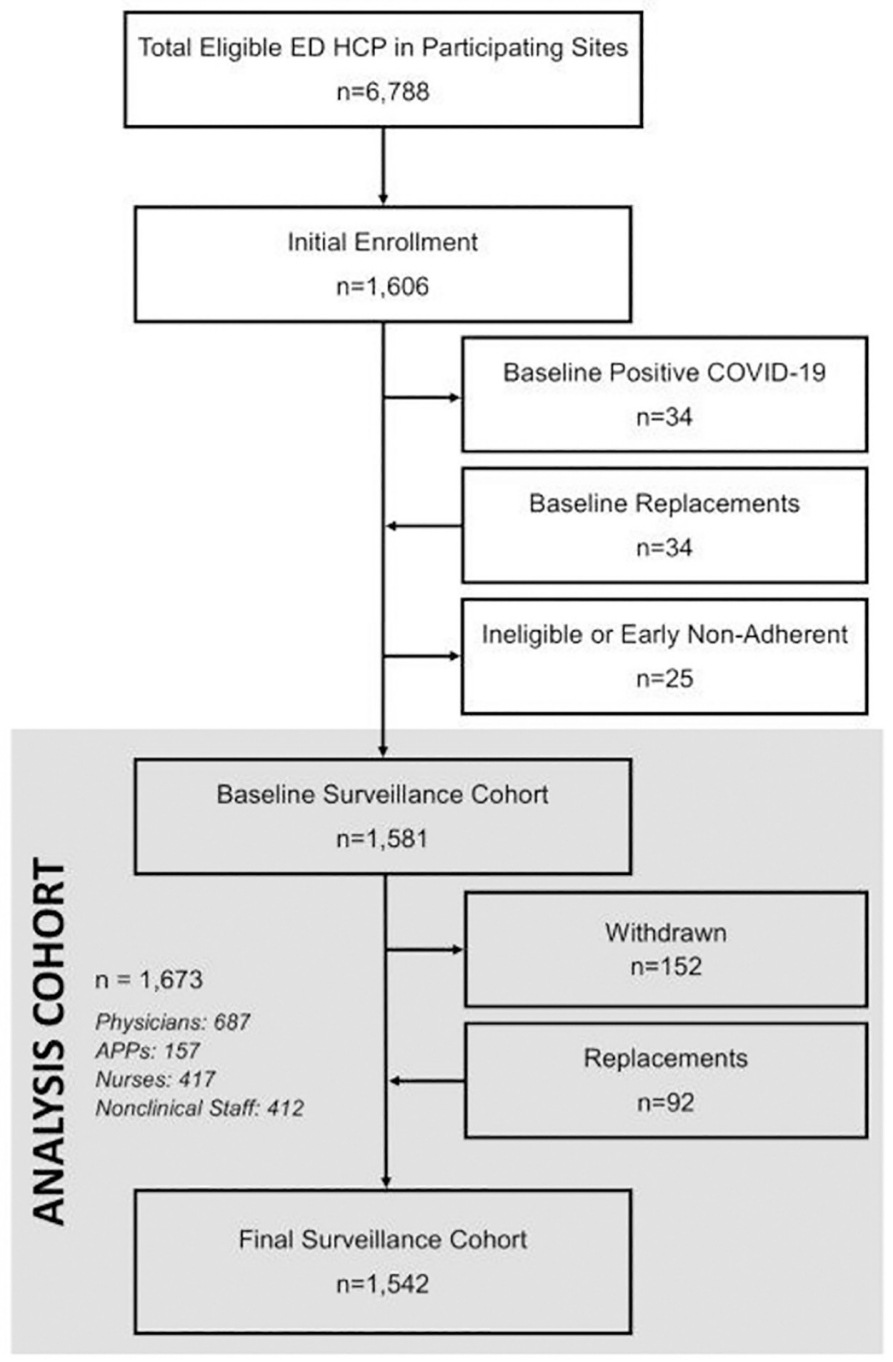
Screening and enrollment. The final analysis cohort had 1,673 participants who contributed 29,825 person-weeks of observation. *Baseline Positive COVID-19* was defined as a positive reverse-transcription polymerase chain reaction (RT-PCR) or a positive SARS-CoV-2 anti-nucleocapsid IgG antibody, and any HCP with a positive baseline test was withdrawn and replaced in the surveillance cohort. *ED*, *emergency department; HCP*, *health care personnel; APP*, *advanced practice provider*.

We enrolled 1,673 ED HCP, who accounted for approximately 21% of all HCP (Fig 1). ED annual visits ranged from 33,000 to 247,519 by site (2019 data). All sites had emergency medicine residency programs (S2 Table). Weekly cumulative community SARS-CoV-2 infection incidence at sites ranged from 5.8 to 31.3 cases per 100,000 population during the study period (S2 Fig). Participants contributed 29,825 person-weeks of observation divided into 8,752 epochs (Table 1, S3 Table). The survey completion rate across all participants and time periods was 89.8%; 91.0% of SARS-CoV-2 blood and nasal samples were collected within four days of the expected date. Physicians/APPs reported 4,439 distinct intubations and cardiac arrests, of which 3,765 (84.8%) patients were tested for SARS-CoV-2. SARS-CoV-2 infection was confirmed in 281 (7.5%) patients, and 53.0% of SARS-CoV-2 positives were unknown at the time of ED treatment.

**Table 1:** Baseline characteristics and time-varying risk of emergency department health care personnel by epoch, United States, May–December 2020.

	*Physicians and Advanced Practice Providers*		*Nurses*		*Non-Clinical Staff*
Characteristic	Intubation or cardiac arrest care during an epoch (n = 1,766 person-epochs)	No intubation or cardiac arrest care during an epoch (n = 2,590 person-epochs)	Intubation or cardiac arrest care during an epoch (n = 956 person-epochs)	No intubation or cardiac arrest care during an epoch (n = 1,256 person-epochs)	(n = 2,171 person-epochs)
**OCCUPATIONAL FACTORS**					
**Job category, n (%)**					
Staff physicians	914 (51.5)	1,110 (42.9)	N/A	N/A	N/A
Resident/Fellows	837 (47.1)	653 (25.2)	N/A	N/A	N/A
APP (PA/NP)	25 (1.4)	827 (3.9)	N/A	N/A	N/A
Nurse	N/A	N/A	956 (100)	1,256 (100)	N/A
Unit clerk/Registration clerk/Financial clerk	N/A	N/A	N/A	N/A	784 (36.4)
Social worker/Case manager	N/A	N/A	N/A	N/A	385 (17.9)
Pharmacist	N/A	N/A	N/A	N/A	137 (6.4)
Other non-clinical staff	N/A	N/A	N/A	N/A	865 (39.8)
**Years since professional school** (physician/APP, nurse), mean (SD)	9.2 (9.3)	10.3 (9.5)	9.2 (7.8)	9.1 (8.0)	N/A
**Work hours per week, mean (SD)**	28.8 (14.7)	24.1 (15.0)	36.1 (10.2)	33.0 (12.4)	32.4 (13.9)
**Other employment in health care, n (%)**	154 (8.7)	320 (12.4)	148 (15.5)	143 (11.4)	129 (5.9)
**DEMOGRAPHICS**					
**Age, median (IQR) years**	33 (30–41)	36 (30–45)	33 (28–41)	34 (28–44)	41 (32–53)
**Gender, n (%)**					
Male	1,138 (64.1)	1,226 (47.3)	196 (20.5)	203 (16.1)	446 (20.5)
Female	634 (35.7)	1,353 (52.2)	751 (78.6)	1,041 (82.7)	1,719 (79.2)
Nonbinary	4 (0.2)	12 (0.5)	9 (0.9)	15 (1.2)	6 (0.3)
**Race, n (%)**					
White	1,514 (85.2)	2,113 (81.6)	822 (8.0)	1,078 (85.6)	1,556 (71.7)
Black	47 (2.6)	144 (5.6)	37 (3.9)	56 (4.4)	342 (15.8)
Asian	195 (11.0)	285 (11.0)	77 (6.4)	78 (6.2)	138 (6.4)
Another race	10 (0.6)	48 (1.9)	20 (2.1)	44 (3.5)	135 (6.2)
**Hispanic or Latino, n (%)**	95 (5.5)	182 (7.3)	83 (9.0)	117 (9.6)	354 (16.7)
**MEDICAL HISTORY**					
**Comorbidities, n (%)**					
Asthma	169 (9.5)	270 (10.4)	107 (11.2)	148 (11.8)	303 (14.0)
Hypertension	115 (6.5)	268 (10.3)	51 (5.3)	73 (5.8)	351 (16.2)
Diabetes mellitus	35 (2.0)	53 (2.0)	21 (2.2)	31 (2.4)	156 (7.4)
Autoimmune disease	77 (4.3)	136 (5.3)	56 (5.9)	49 (3.9)	119 (5.5)
Immune Suppression	43 (2.4)	33 (1.3)	48 (5.0)	26 (2.1)	92 (4.2)
**Smoking, n (%)**					
Current smoker	7 (0.4)	13 (0.5)	63 (6.6)	81 (6.4)	165 (7.6)
Former smoker	92 (5.2)	195 (7.5)	125 (13.1)	25 (19.9)	444 (20.5)
Never smoker	1,645 (92.6)	2,334 (90.1)	748 (78.2)	908 (72.1)	1,489 (68.6)
**RISK FACTORS, n (%)**					
CDC-adherent workplace PPE use (routine care)	1767 (99.5)	2577 (99.5)	954 (99.8)	1251 (99.4)	2171 (100)
CDC-adherent workplace PPE use (COVID-19 care)	1775 (99.9)	2589 (100)	956 (100)	1255 (99.7)	2171 (100)
CDC-adherent workplace PPE Use (COVID-19 aerosol-generating procedures)	1775 (99.9)	2587 (99.9)	956 (100)	1255 (99.7)	2171 (100)
Workplace PPE shortages	700 (39.4)	1032 (39.9)	293 (30.7)	416 (33.0)	2171 (100)
Workplace PPE re-use	1439 (81.0)	2101 (81.1)	727 (76.1)	1060 (84.2)	0 (0)
Household COVID-19 exposure	10 (0.6)	5 (0.2)	5 (0.5)	12 (1.0)	14 (0.6)
Attended mass gatherings	411 (23.1)	490 (18.9)	259 (27.1)	326 (25.9)	416 (19.2)
Used public transportation	249 (14.0)	353 (13.6)	144 (15.1)	190 (15.1)	268 (12.3)
Personal mask use in community	954 (53.7)	1454 (56.1)	462 (48.3)	587 (46.6)	1554 (71.6)
Hospital COVID-19 cases (>100/week)	65 (3.7)	103 (4)	32 (3.4)	56 (4.5)	72 (3.3)
Community cumulative COVID-19 incidence (>15/100,000 population)	513 (28.9)	479 (18.5)	248 (25.9)	248 (19.7)	528 (24.3)
Weekly COVID-19 patients treated per week, mean (SD)	1.5 (0.9)	1.1 (0.8)	1.3 (0.7)	1.1 (0.8)	0 (0)
Hospital COVID-19 cases, mean (SD)	27.6 (28.3)	25 (29.1)	28.4 (28.3)	24.9 (29.1)	27.1 (28.5)
Community cumulative COVID-19 Incidence, mean (SD)	12.7 (10.8)	10.5 (9.3)	13 (12.5)	10.7 (8.5)	12.1 (11.5)

Emergency department (ED) health care personnel (HCP) are stratified by time-epoch. Participation in intubation or cardiac arrest care required that the HCP be within three feet of the patient’s mouth during intubation or cardiac arrest care. Because some risk categories are time-varying, participants may be included in different categories for different time periods. *Other employment in healthcare* means that the participant had another healthcare job in a non-COVERED site. *APP*, *advanced practice provider; PA*, *physician assistant; NP*, *nurse practitioner; SD*, *standard deviation; N/A*, *not applicable*.

### Personal protective equipment use and risk

Based on weekly surveys, clinical staff commonly reported masking at all times while working in the ED (96.9% of person-weeks). HCP used surgical masks routinely in 18,009 (80.5%) person-weeks for non-clinical activities and 17,785 (79.5%) person-weeks for non- SARS-CoV-2 infection patient care. For care of COVID-19 patients, N95 (or higher) masks were used routinely in 18,836 (85.4%) person-weeks and in 19,396 (86.7%) person-weeks during an aerosol-generating procedure (S3 Fig). Clinical HCP reported lapses in SARS-CoV-2 infection PPE use (defined as caring for a SARS-CoV-2 patient without at least a surgical mask and gloves) in 4,944 (22.1%) person-weeks.

**Fig 2:**
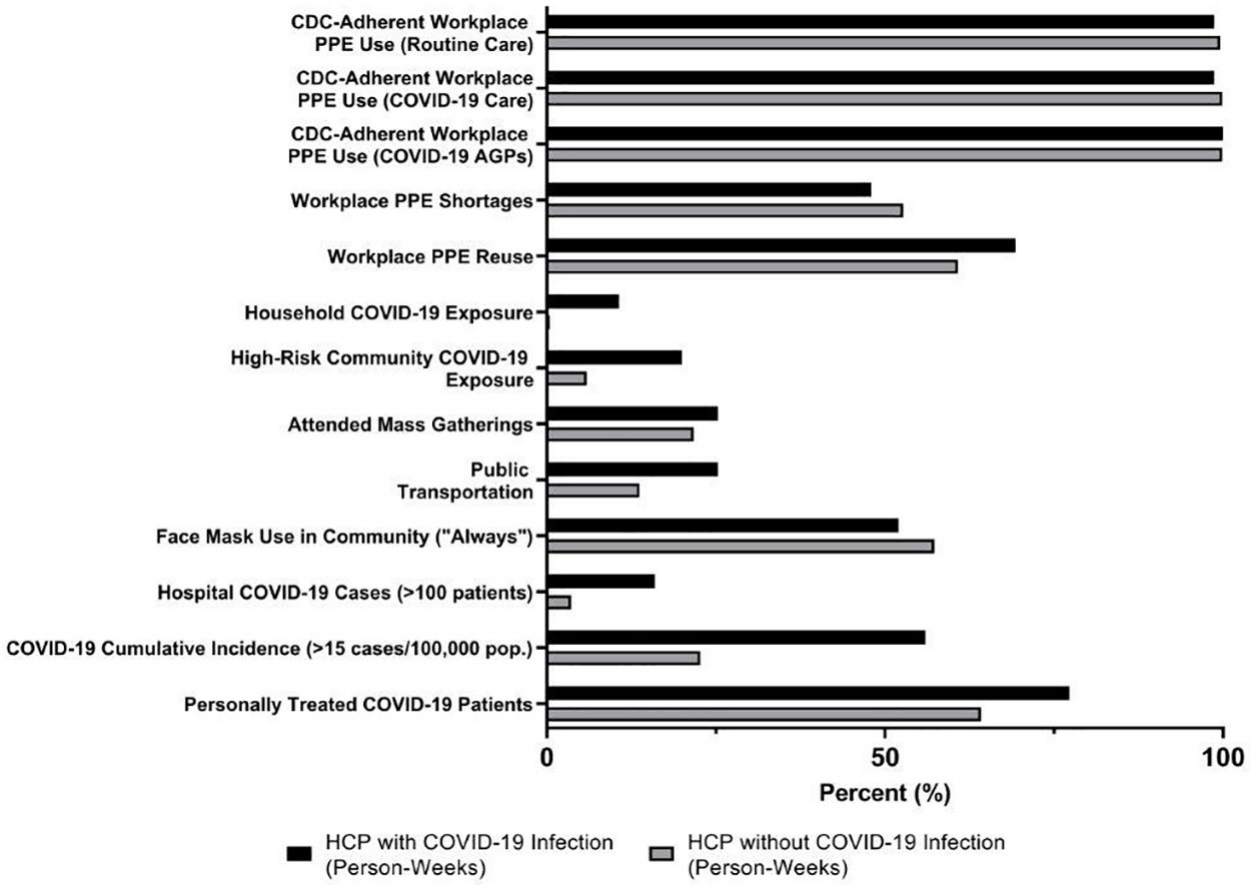
Occupational and community COVID-19 infection risk in emergency department health care personnel, United States, May–December 2020. This graph shows the percentage of participant epochs with each of the a priori-defined risk factors. Risk factor definitions are summarized in S1 Table. *CDC*, *Centers for Disease Control and Prevention*, *CDC; AGP*, *aerosol-generating procedures; PPE*, *personal protective equipment; HCP*, *health care personnel*.

In the procedure-level analysis of physicians/APPs SARS-CoV-2 patient intubations or CPR, 314 (90.8%) of 346 HCP used N95 masks or powered air-purifying respirators (PAPR), 293 (84.7%) used eye protection, and 294 (85.0%) used gowns. Guideline-adherent PPE (gown, eye protection, and N95 mask or PAPR) use occurred in 235 events (67.9%) and was not higher when the care team knew a patient had SARS-CoV-2 infection prior to the procedure (68.1% vs. 69.3%; OR 0.97; 95% CI, 0.60–1.54). Adherence with CDC-recommended PPE use and community exposures are shown in Fig 2.

Twenty-four (96%) of the 25 EDs had periods in which at least one participant reported inadequate PPE and, in all facilities staff reused single-use PPE because of supply shortages (S2 Table). Outside of work, participants commonly reported (69.2% of person-weeks) universal masking in public (S4 Table).

### ED HCP risk of acquiring SARS-CoV-2 infection

During the 20-week surveillance period, 75 (4.5%) HCP developed SARS-CoV-2 infection, with site-specific incidence ranging from 0% to 9.4%. SARS-CoV-2 infection developed in 29 of 417 (7.0%; 95% CI, 48% –10.1%) nurses, 31 of 844 (3.7%; 95% CI, 2.3% –6.0%) physicians/APPs, and 15 of 412 (3.7%; 95% CI, 2.0% –6.8%) non-clinical ED HCP. Among those infected, 31 (41%) remained asymptomatic. Characteristics of HCP who developed SARS-CoV-2 infection are listed in S5 Table.

### HCP COVID-19 risk factors

**Fig 3:**
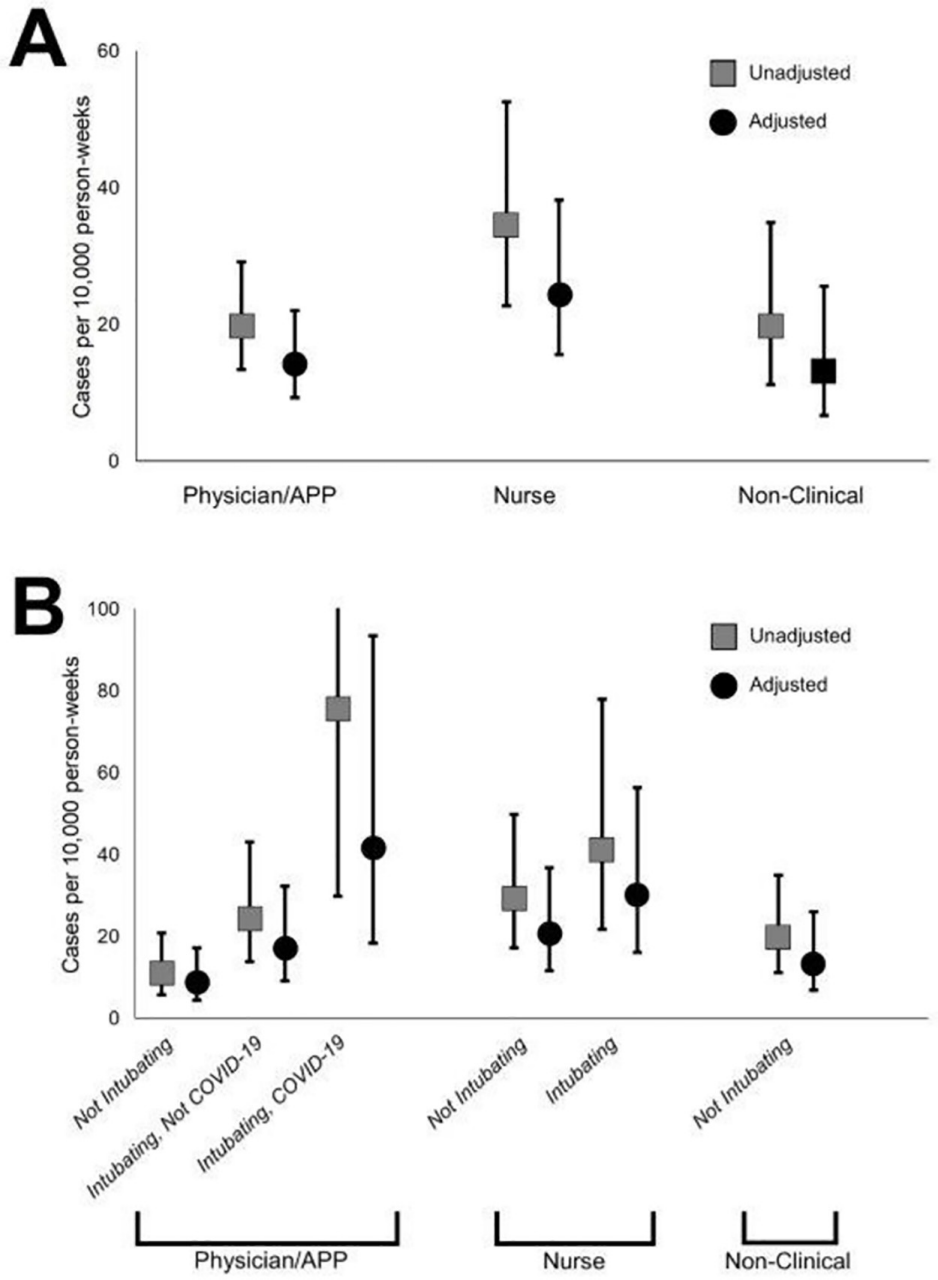
Adjusted COVID-19 infection risk in emergency department health care personnel stratified by job type and intubation, United States, May–December 2020. These graphs show the unadjusted and adjusted attributable risk point estimates, and error bars show the upper and lower bounds of the 95% confidence intervals. Adjusted estimates account for household and community COVID-19 exposure, number of in-hospital COVID-19 cases, and community COVID-19 incidence. **A**. Risk of HCP COVID-19 infection shown within each of three job categories. **B**. Risk of HCP COVID-19 infection shown within categories of risk and intubation/cardiac arrest care risk assigned within each participant time-epoch. Note that risk was assigned at the epoch-level, so one participant may have contributed epochs in the intubation risk category when intubations were performed and in the non-intubation risk category when no intubations were performed. *Not intubating* represents the risk in epochs during which no intubation or cardiac arrest care was reported. *Intubating*, *not COVID-19* represents the risk in epochs during which only non-COVID-19 intubations or cardiac arrest care was reported. For nurses, all intubation and cardiac arrest care is represented in this category since no stratification was performed. *Intubating*, *COVID-19* represents risk in epochs during which any RT-PCR-confirmed COVID-19 patients had intubation or cardiac arrest care. *APP*, *advanced practice provider*. *cases per 10*,*000 person-weeks*, *number of COVID-19 infections identified per 10*,*000 person-weeks of observation*.

In our multivariable regression model, the following epoch factors were independently associated with COVID-19 infection: 1) household SARS-CoV-2 exposure (aOR 16.34; 95% CI, 5.79–46.09); 2) hospital SARS-CoV-2 case count (aOR 3.28; 95% CI, 1.68–6.40); 3) community SARS-CoV-2 infection cumulative incidence (aOR 3.21; 95% CI 1.95–5.29); and 4) community SARS-CoV-2 exposure (aOR 2.38; 95% CI 1.24–4.54) (S6 Table). Adjusted odds ratios for physicians (aOR 1.07; 95% CI, 0.56–2.03) and nurses (aOR 1.91; 95% CI, 0.99–3.68) relative to non-clinical staff did not achieve statistical significance (Table 2). Using recursive partitioning, we found the following factors associated with COVID-19 infection: 1) hospital SARS-CoV-2 volume; 2) community SARS-CoV-2 infection cumulative incidence; and 3) lack of personal mask use outside of work, and these 3 factors explained 63 (84.0%) of HCP infections (S7 Table). Adjusted for identified risk factors in our regression model, risk of SARS-CoV-2 infection stratified by job category is described in Fig 3. Occupational PPE use was not significantly associated with infection.

**Table 2:** Unadjusted and adjusted COVID-19 infection risk in emergency department health care personnel by exposure type, United States, May–December 2020.

Risk Factor	Comparator group	Percentage with risk factor who developed COVID-19 (no. of epochs with risk factor/total epochs at risk, %)	Percentage in comparator group who developed COVID-19 (no. of epochs without risk factor/total epochs at risk, %)	Unadjusted OR (95% CI)	Adjusted OR (95% CI)	Adjusted Attributable Fraction (95% CI)
Physician/APP	Nonclinical Staff	31/4366, 0.71%	15/2171, 0.69%	1.03 (0.55–1.91)	1.07 (0.56–2.03)	4.3% (-41.5%, 40.7%)
Physician/APP who intubated during the study	Physician/APP who did not intubate during the study	23/3169, 0.73%	8/1197, 0.67%	1.09 (0.49–2.45)	1.09 (0.47–2.56)	6.4% (-62.9%, 53.1%)
Physician/APP who intubated during a risk epoch	Physician/APP who did not intubate during a risk epoch	20/177 9, 1.12%	11/2590, 0.42%	2.66 (1.26–5.61)	2.17 (1.02–4.62)	32.2% (0.7%, 59.6%)
Physician/APP who intubated an RT-PCR-confirmed COVID-19 patient during the study	Physician/APP who intubated a patient during the study but did NOT intubate an RT-PCR-confirmed COVID-19 patient during the study	9/1038, 0.87%	14/2131, 0.66%	1.33 (0.58–3.07)	0.91 (0.40–2.09)	-3.1% (-24.7%, 26.3%)
Physician /APP who intubated an RT-PCR-confirmed COVID-19 patient during a risk epoch	Physician/APP who intubated a patient during a risk epoch but did not intubate an RT-PCR-confirmed COVID-19 patient during that risk epoch	6/243, 2.47%	14/1533, 0.91%	2.77 (1.04–7.36)	2.17 (0.85–5.51)	13.8% (-2.0%, 38.2%)
Nurse	Nonclinical Staff	29/2215, 1.31%	15/2171, 0.69%	1.92 (1.03–3.57)	1.91 (0.99–3.68)	31.6% (-0.3%, 57.5%)
Nurse who was involved with intubation during the study	Nurse who was not involved with intubation during the study	24/1681, 1.43%	5/534, 0.94%	1.54 (0.59–4.07)	1.52 (0.57–4.07)	28.2% (-49.1%, 70.0%)
Nurse who was involved with intubation during a risk epoch	Nurse who was not involved with intubation during a risk epoch	13/956, 1.36%	16/1259, 1.27%	1.07 (0.51–2.25)	1.10 (0.52–2.32)	4.0% (-26.2%, 36.3%)

Risk of developing COVID-19 was adjusted for hospital COVID-19 volume, community COVID-19 cumulative incidence, household COVID-19 exposure, and high-risk community COVID-19 exposure. For study-level exposures, participants were assigned to a risk group if they intubated during the entire 20-week study and, for epoch-level exposures, participants were assigned only if they intubated during the risk epoch. *OR*, *odds ratio; 95% CI*, *95% confidence interval*.

### Aerosol-generating procedure risk

SARS-CoV-2 infection developed in 9 (4.4%) of 205 physicians/APPs who performed intubation or cardiac arrest care for a SARS-CoV-2 patient and 22 (3.4%) of 639 physicians/APPs who did not perform these procedures for a SARS-CoV-2 patient (difference 0.9%; 95% CI, -2.7–4.6%). Adjusting for other risk factors, SARS-CoV-2 infection risk was not higher for physicians/APPs in the relevant epoch after they intubated a SARS-CoV-2 patient compared with physicians/APPs who intubated only patients without SARS-CoV-2 infection (AF 13.8%; 95% CI, -2.0–38.2%), but it was higher than non-clinical HCP (aOR 2.75, 95% CI 1.08–6.99). Physicians/APPs performing intubations during risk epochs (versus not performing intubations) had higher infection risk (AF 32.2%; 95% CI, 0.7–59.6%) (Table 2, S6 Table). Six of 31 total SARS-CoV-2-infected physicians/APPs intubated or provided cardiac arrest care for a SARS-CoV-2 patient in a relevant epoch, including two who performed this care without guideline-adherent PPE (S5 Table). SARS-CoV-2 infection was not associated with intubation or cardiac arrest care among participating nurses (AF 4.0%; 95% CI, -26.2–36.3%). The sensitivity analysis excluding intubation or cardiac arrest patients without SARS-CoV-2 testing revealed similar results.

## Discussion

Over 20 weeks of prospective surveillance of high-risk ED HCP with routine recommended PPE use during the height of COVID-19 pandemic in 2020, prior to vaccine availability, and prior to emergence of the Omicron variant, we identified the strongest risks for SARS-CoV-2 infection. These included household and community exposures (people in the home or community with a respiratory illness suspected or confirmed to be SARS-CoV-2 infection), hospital case count, and mask non-use in public. Intubation and cardiac arrest care of SARS-CoV-2 patients appeared to be associated with an incremental risk of SARS-CoV-2 infection, but the low frequency of these events did not translate into detectable increased risk for HCP during the surveillance period. Our finding that nonclinical staff had infection rates similar to clinical HCP is consistent with risk primarily from community and hospital exposure as opposed to direct patient care, including performing aerosolizing procedures. However, it is important to note that nurses still had the highest absolute SARS-CoV-2 infection risk.

Even though SARS-CoV-2 vaccination is now available, low worldwide vaccination coverage, increasing concern about novel SARS-CoV-2 variants, and waning vaccination immunity suggest that infection prevention measures among HCP remain critical. ED HCP have a high risk of SARS-CoV-2 exposure and many remain unvaccinated [1, 2, 24]. Our findings align with prior work, suggesting that HCP who use recommended PPE within the context of a comprehensive infection prevention program can effectively protect themselves from occupational SARS-CoV-2 infection [25, 26].

HCP have been previously identified as a high-risk cohort, but the impact of occupational exposure, including performing high-exposure procedures, may have been attenuated with increasing familiarity with precautions and PPE use [25, 27, 28]. Emergency intubation was recognized as a high-risk exposure for nosocomial SARS-1 transmission (aOR 2.79; 95% CI 1.40–5.58) during the Toronto outbreak in 2003, and it is widely thought to confer risk of SARS-CoV-2 transmission [29–31]. In an analysis of the international *intubateCOVID* registry using self-reported data, SARS-CoV-2 infection was identified in 8.5% of HCP within 21 days after their first reported intubation procedure, and transmission was unrelated to PPE use [8]. Our data suggest that this risk, if present, is very small among a cohort that frequently used airborne, contact, and droplet precautions.

Our findings suggest that the greatest infection risk may not arise from specific procedures or from clinical care when using appropriate infection control measures and PPE; it may come from being in an environment with patients, family members, and staff infected with SARS-CoV-2. We showed previously, and confirmed in this study, that HCP with SARS-CoV-2 were often asymptomatic, which has substantial relevance to PPE use and staff-to-staff infection mitigation strategies [5]. In fact, staff-to-staff transmission has previously been shown to be an important route of HCP transmission [32]. SARS-CoV-2 can propagate efficiently through the hospital environment [33]. Among hospitalized patients with asymptomatic SARS-CoV-2 infected roommates, nearly 40% contracted SARS-CoV-2 infection after a median of only 18 hours of exposure [6]. Furthermore, the risk to HCP from work is not just occupational risk. During the period of the study, it also included exposures due to commuting, being in a hospital environment where social distancing is difficult, and PPE shortages, which were reported in most site hospitals—all risks borne by clinical and nonclinical ED staff. While data collection was completed prior to the widespread COVID-19 vaccine availability, this non-occupational risk suggests that community and COVID-19 vaccination and mitigation strategies may play important roles in protecting HCP and their patients and helping to keep healthcare systems functional during a pandemic [34].

Although we found nurses to have the highest absolute SARS-CoV-2 infection risk, the relative risk did not reach statistical significance. Nurses have previously been shown to commonly acquire SARS-CoV-2 infection [7, 9, 35, 36]. Nurses have higher cumulative time at the bedside, which may contribute to risk—a factor identified in nurses who treated SARS-1 patients during the Toronto outbreak [37].

Finally, there were frequent lapses in occupational PPE use, and community mask use was imperfect. HCP worldwide during this period redefined their longstanding work practices based on changing guidance for personal protection. Barriers to PPE use, shortages, and uncertainty around re-use have previously been identified [38]. These frequent lapses may justify revised PPE procedures and training from public health authorities and employers to improve HCP safety in future outbreaks.

This study has limitations. Although we had high study procedure adherence, we relied on self-reported exposure data. Fifteen percent of intubation and cardiac arrest patients were not tested for SARS-CoV-2, allowing some misclassification. We enrolled HCP from diverse clinical settings, but all were at academic centers. Our non-clinical population was used to approximate the risk of clinical care, but they may have had higher or lower risk than the general population. We enrolled our planned sample, but our assumptions underestimated the burden of SARS-CoV-2 infection in our cohort. We used explanatory modeling alone to identify factors associated with SARS-CoV-2 transmission, leaving the possibility of overfitting in our models, but we used only a priori-defined factors and limited our variables to improve reproducibility of our findings. Finally, the highly infectious SARS-CoV-2 Delta (B.1.617.2) and Omicron (BA.1, BA.1.1, BA.2) variants were not present at the time of our study.

In conclusion, risk of SARS-CoV-2 infection was similar in nonclinical staff and ED HCP engaged in direct clinical care during the height of the COVID-19 pandemic in the United States in 2020, despite frequent exposure of clinical ED HCP to high-risk aerosol-generating procedures. The most important infection risk factors affect both nonclinical staff and clinical HCP and include household and community exposures, hospital case counts, and mask non-use in public. PPE availability and use were generally good but imperfect, and infections were often asymptomatic. This work reinforces the importance of universal HCP vaccination and ongoing community mitigation and vaccination strategies. Future work should focus on comprehensive prevention strategies to maintain PPE availability to ensure a healthy HCP workforce, protect patients, and maintain adequate health system capacity.

## Supporting information

S1 Fig Definition of time epochs used for risk factor analysis. (PDF)

S2 Fig SARS-CoV-2 activity at participating centers. (PDF)

S3 Fig Personal protective equipment use in emergency departments, percentage by person-week over study period. (PDF)

S1 Table Detailed definitions of candidate risk factors for sars-cov-2 infection in emergency department health care personnel. (PDF)

S2 Table Characteristics of participating emergency departments. (PDF)

S3 Table Baseline characteristics of participants. (PDF)

S4 Table Non-occupational exposures of participants by job category. (PDF)

S5 Table Characteristics of participants who developed SARS-CoV-2 infection during the risk epoch. (PDF)

S6 Table Multivariable regression models for risk factor analysis. (PDF)

S7 Table Recursive partitioning analysis. (PDF)

S1 File COVID Evaluation of Risk for Emergency Departments (COVERED) project: Project protocol. (PDF)

S2 File Supplemental statistical procedure. (PDF)
