# Supplementary material for: Emergency department personnel patient care-related COVID-19 risk
Source: PLoS One. 2022 Jul 22;17(7):e0271597. doi: 10.1371/journal.pone.0271597 (PMC9307202; doi:10.1371/journal.pone.0271597)
Supplement: S1 Fig — (PDF) [file pone.0271597.s001.pdf]

S1 Fig. Definition of Time Epochs Used for Risk Factor Analysis

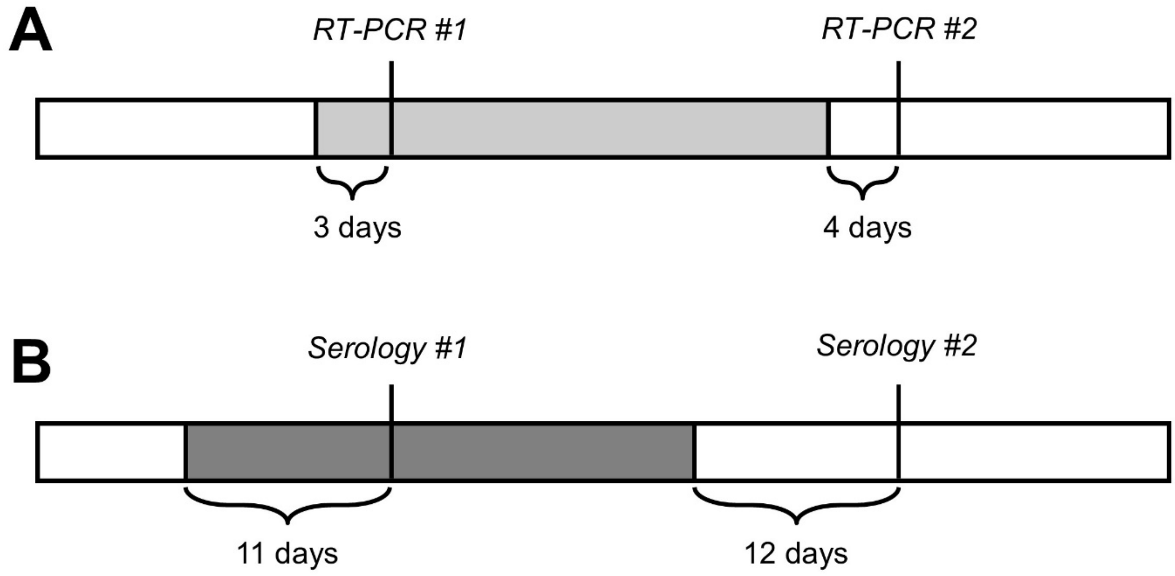

We defined time epochs based on reverse-transcription polymerase chain reaction (RT-PCR) and serology testing, including a standard lag for time to positive testing. We defined a RT-PCR-based epoch as the period from three days before the *previous* RT-PCR test to four days before the *current* test (to maintain mutually exclusive consecutive time epochs), while a corresponding threshold of 12 days was used for serology results. We used RT-PCR positive results to define the risk epoch unless no RT-PCR was positive or the serology was positive before the RT-PCR (in which case a serology-based epoch was used). In the example shown in the figure, the risk period shown would correspond with the results of RT-PCR #2 or Serology #2. **A.** RT-PCR based epoch definition. **B.** Serology based epoch definition.
