# Supplementary material for: Emergency department personnel patient care-related COVID-19 risk
Source: PLoS One. 2022 Jul 22;17(7):e0271597. doi: 10.1371/journal.pone.0271597 (PMC9307202; doi:10.1371/journal.pone.0271597)
Supplement: S2 Fig — (PDF) [file pone.0271597.s002.pdf]

S2 Fig. SARS-CoV-2 Activity at Participating Centers

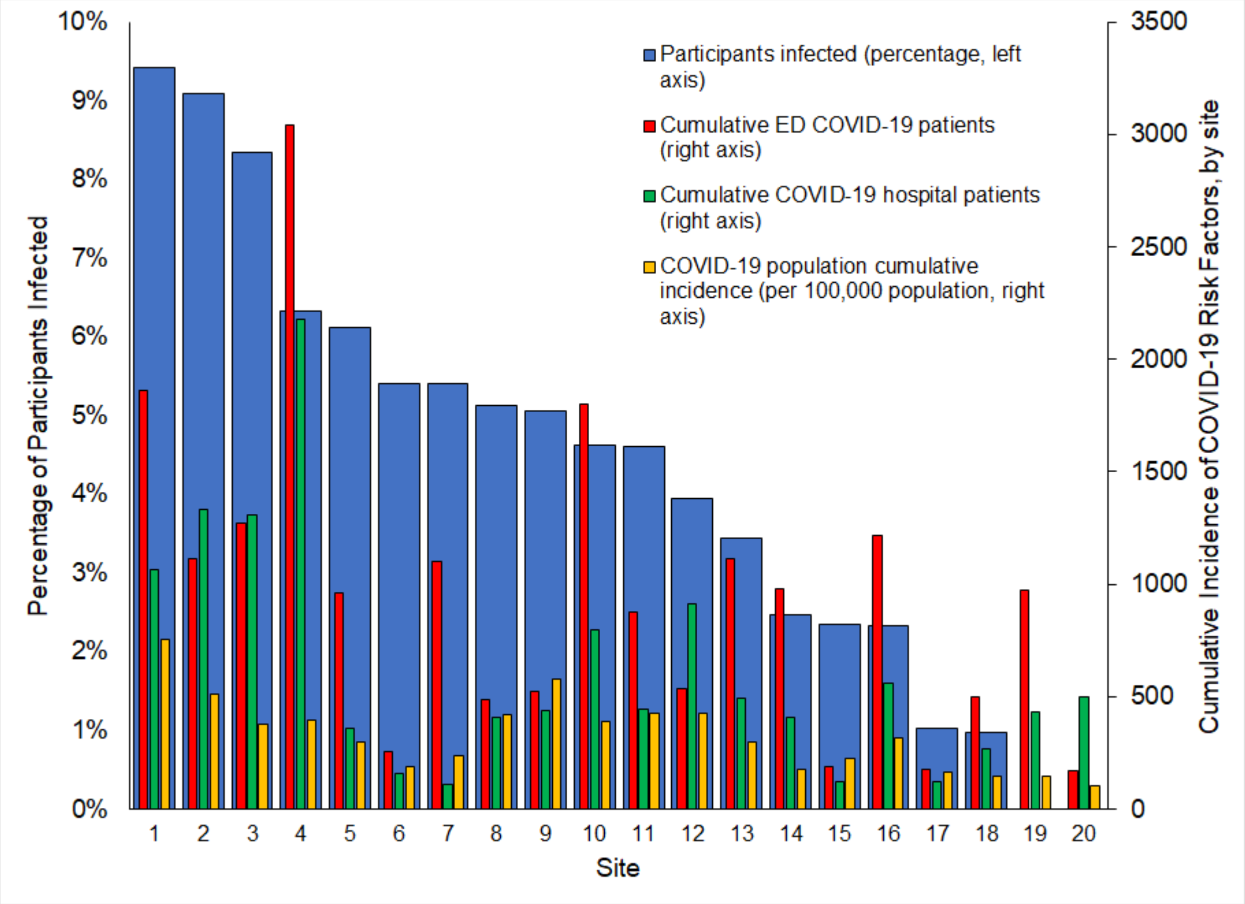

This graph shows the relationship between site-specific COVID-19 infections among participating health care personnel and site-specific exposures. Each site is represented by a cluster of bars. The **blue bars** show the percentage of participants developing COVID-19 at each site (left vertical axis). The **red bars** represent the cumulative number of COVID-19 patients treated in the ED during the period of surveillance (right vertical axis). The **green bars** represent the cumulative number of COVID-19 patients admitted to participating hospitals during the period of surveillance (right vertical axis). The **orange bars** represent the cumulative incidence of COVID-19 in the health service area of the participating hospital, from public health reports, during the period of surveillance (right vertical axis).
