# Supplementary material for: Emergency department personnel patient care-related COVID-19 risk
Source: PLoS One. 2022 Jul 22;17(7):e0271597. doi: 10.1371/journal.pone.0271597 (PMC9307202; doi:10.1371/journal.pone.0271597)
Supplement: S3 Fig — (PDF) [file pone.0271597.s003.pdf]

S3 Fig. Personal Protective Equipment Use in Emergency Departments, Percentage by Person-Week over Study Period

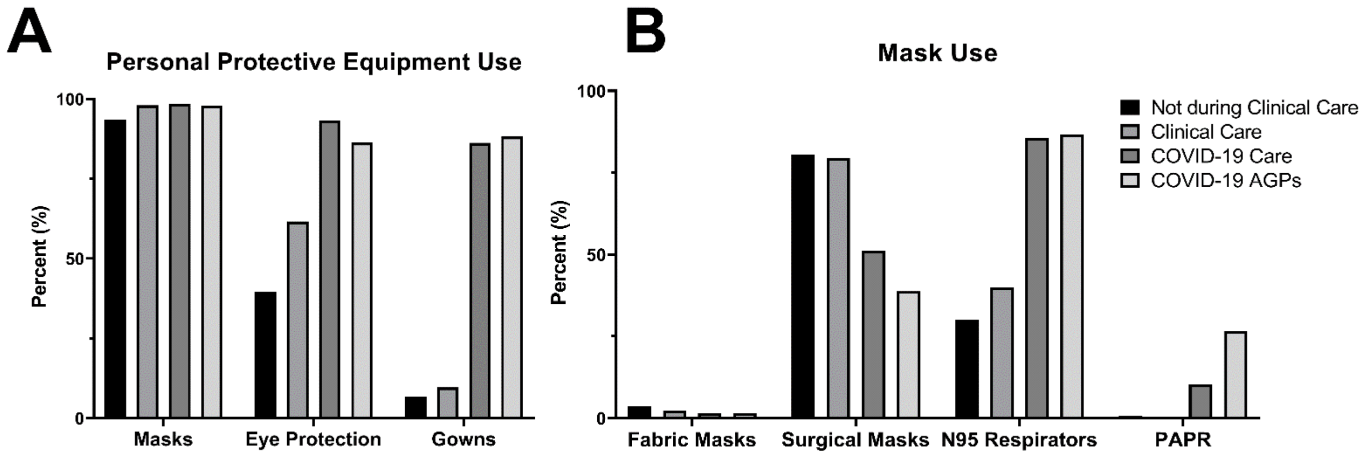

Participants reported on weekly survey forms what personal protective equipment they used routinely for activities in the ED (*Not during clinical care*, *clinical care* of patients not known or suspected to have COVID-19, *COVID-19 clinical care*, *clinical care* while performing aerosol-generating procedures [*AGPs*] in COVID-19 patients). Percentages on these graphs reflect person-weeks of observation. **A.** Personal protective equipment use. **B.** Types of masks used for clinical care. *AGP, aerosol-generating procedures; PAPR, powered air-purifying respirator.*
