# Supplementary material for: Emergency department personnel patient care-related COVID-19 risk
Source: PLoS One. 2022 Jul 22;17(7):e0271597. doi: 10.1371/journal.pone.0271597 (PMC9307202; doi:10.1371/journal.pone.0271597)
Supplement: S1 Table — (PDF) [file pone.0271597.s004.pdf]

S1 Table. Detailed Definitions of Candidate Risk Factors for SARS-CoV-2 Infection in Emergency Department Health Care Personnel.

Factor	Detailed Description
1	**Weekly number of COVID-19 patients receiving care from an individual provider.** In weekly surveys we asked health care providers (HCP) to indicate the number of COVID-19 patients they had cared for using the following classifications:<br>1 – None;<br>2 - One to five;<br>3 - Six to ten; or<br>4 - More than ten.<br>For risk factor analysis, we identified the most predictive threshold empirically for testing.
2	**Weekly hospital volume of COVID-19 patients.** Using weekly data provided by each participating facility, we categorized the weekly hospital volume of COVID patients into one the following five strata:<br>1 - 25 patients or fewer;<br>2 - 50 patients or fewer;<br>3 - 75 patients or fewer;<br>4 - 100 patients or fewer; or<br>5 - Greater than 100 patients.<br>For risk factor analysis, we identified the most predictive threshold empirically for testing.
3	**Cumulative community incidence of COVID-19 infections.** Using weekly data from the CDC health services area associated with each of the participating centers, we categorized the weekly community volume of COVID infected people into one of the following four strata:<br>1 – 8·0 per 100,000 or less;<br>2 – 15·0 per 100,000 or less;<br>3 – 30·0 per 100,000 or less; or<br>4 - Greater than 30·0 per 100,000 population.<br>For risk factor analysis, we identified the most predictive threshold for additional analysis.
4	**Provider age.** Using data from baseline surveys, we assigned providers into one of the following four age groups:<br>1 - ≤ 30·0 years;<br>2 – 30·0 years < age ≤ 40·0 years;<br>3 – 40·0 years < age ≤ 50·0 years;<br>4 - >50·0 years old.
5	**Provider type.** Based on provider responses, we classified HCP into one of the following four groups:<br>1 - Intubating physician/APP;<br>2 - Non-intubating physician/APP;<br>3 - Nurse; or<br>4 - Non-clinical care provider.
6	**Provider specialty:** Based on provider responses in the baseline survey, we classified physicians/APPs into one of the following five groups:<br>1 - Emergency Department Attending;<br>2 - Emergency Department Resident;<br>3 - Advanced Practice Provider;<br>4 - Non-Emergency Department Attending; or<br>5 - Non-Emergency Department Resident.
7	**Use of personal protective equipment (PPE) when providing routine patient care.** Based on provider responses from weekly surveys, we classified providers as low-risk PPE use if they performed standard hygiene precautions, used eye protection, and wore a mask when providing routine care for non-COVID-19 patients. We assigned high-risk status to providers who did not meet requirements for low-risk classification.
8	**Use of PPE when providing care for COVID-19 patients.** Based on provider responses from weekly surveys, we classified providers as low-risk PPE use if they performed standard hygiene precautions, employed eye protection, wore a gown and gloves, and wore an N95 mask, elastomeric respirator or powered air-purifying respirator (PAPR, we considered surgical and fabric masks inadequate) when providing routine care for COVID-19 patients. We assigned high-risk status to providers who did not

	meet requirements for low-risk classification.
9	**Use of PPE when performing high-risk procedures.** Based on provider responses from weekly surveys, we classified providers as low-risk PPE use if they performed standard hygiene precautions, employed eye protection, wore a gown and gloves, and wore an N95 mask, elastomeric respirator or PAPR (we considered surgical and fabric masks inadequate) when performing high-risk procedures, such as intubation and cardiac arrest care, on COVID-19 patients. We assigned high-risk status to providers who did not meet requirements for low-risk classification.
10	**Institutional availability of PPE.** Based on provider responses from weekly surveys, we classified providers as low-risk in terms of institutional availability of PPE use if they had sufficient resources to perform standard hygiene precautions, had access to protective eye wear, gowns, gloves, and N95 masks, elastomeric respirators or PAPRs. We assigned high-risk status to providers who did not meet requirements for low-risk classification.
11	**Household exposure to confirmed COVID-19 infection.** Based on responses from weekly surveys, we assigned HCPs as having high-risk for household exposures if they met the following criteria: Slept at home and experienced close contact with another person who had a confirmed COVID-19 test, or had symptoms of COVID-19 with suspected COVID-19 infection during the preceding week. We assigned low risk status to providers who did not meet requirements for high-risk classification.
12	**Public exposure to suspected COVID-19 cases.** Using data from weekly surveys, we classified providers as having high-risk public exposure from friends or community if they indicated they had contact with a friend or community member with symptoms of COVID-19 infection during the preceding week. We classified providers as low-risk if they had no contact with friends or community members who had symptoms of COVID-19.
13	**Public exposure from crowds, mass gatherings, transportation, or travel.** Using data from weekly surveys, we classified providers as having low-risk exposure to crowds, mass gatherings, transportation, or travel if they indicated they had not participated in any crowds or mass gatherings, had not used public transportation, or travelled in crowds during the preceding week. We classified providers as high-risk if they did not meet low-risk classification.
14	**Use of face mask in public.** Based on responses to weekly surveys, we classified providers as low-risk if they consistently used face coverings in public during the preceding week. We classified providers as high-risk if they did not consistently use face coverings in public.
15	**Performing high-risk procedures.** We classified providers as performing of high-risk procedures if they submitted one or more high-risk procedure reports in conjunction with any weekly survey. We classified providers as not performing high-risk procedures if they never submitted a high-risk procedure report.

Risk factors were assigned for each epoch, so some risk factors changed for a single participant over time. Some continuous variables were stratified into overlapping dichotomous categories, and the candidate cut point was selected that best discriminated between cases and non-cases. *Standard Precautions* included hand washing and maintaining minimum six feet distance from patients. *Adequate eye protection* included reusable face shields, disposable face shields or safety glasses or goggles. *Adequate masks* for routine care included surgical masks, reusable fabric masks, N95 masks and respirators, and powered air-purifying systems. High-risk procedures included endotracheal intubation, cardiopulmonary resuscitation, or combined intubation and cardiopulmonary resuscitation. *HCP, health care personnel; CDC, Centers for Disease Control and Prevention; PPE, personal protective equipment.*
