# Supplementary material for: Emergency department personnel patient care-related COVID-19 risk
Source: PLoS One. 2022 Jul 22;17(7):e0271597. doi: 10.1371/journal.pone.0271597 (PMC9307202; doi:10.1371/journal.pone.0271597)
Supplement: S2 Table — (PDF) [file pone.0271597.s005.pdf]

S2 Table. Characteristics of Participating Emergency Departments

Characteristic	Emergency Departments (n = 25)
2019 ED visits, median (IQR)	73,000 (64,247–94,373)
Emergency medicine residency program, n (%)	25 (100)
ED beds pre-COVID-19, median (IQR)	68 (50-82)
Number of hours staff physician coverage weekly, median (IQR)	504 (365 – 672)
Clinical Operations	
COVID-19 patients are cohorted in a dedicated space/region in the ED, n (%)	14 (56)
ED intubation team was used, n (%)	3 (12)
PPE	
Inadequate PPE reported by at least 1 HCP during at least 1 week, n (%)	24 (96)
Duration of inadequate PPE (weeks), median (IQR)	17 (14–20)
Reuse of single-use PPE routinely reported by at least 1 HCP during at least 1 week, n (%)	25 (100)
Duration of PPE reuse (weeks), median (IQR)	20 (20–20)

All participating EDs are included in this table, including 25 EDs affiliated with 20 academic medical centers. *IQR, interquartile range; PPE, personal protective equipment; HCP, health care personnel.*
