# Supplementary material for: Emergency department personnel patient care-related COVID-19 risk
Source: PLoS One. 2022 Jul 22;17(7):e0271597. doi: 10.1371/journal.pone.0271597 (PMC9307202; doi:10.1371/journal.pone.0271597)
Supplement: S3 Table — (PDF) [file pone.0271597.s006.pdf]

S3 Table. Baseline Characteristics of Participants.

Characteristic	Physicians and APPs (n = 844)	Nurses (n=417)	Non-Clinical Staff (n=412)
**OCCUPATIONAL FACTORS**			
**Job Category**			
Staff physicians, n (%)	376 (44·6)	N/A	N/A
Resident/fellows, n (%)	311 (36·9)	N/A	N/A
APP (PA/NP), n (%)	157 (18·6)	N/A	N/A
Nurse, n (%)	N/A	417 (100·0)	
Unit clerk/registration clerk/financial clerk, n (%)	N/A	N/A	149 (36·2)
Social worker/case manager, n (%)	N/A	N/A	71 (17·2)
Pharmacist, n (%)	N/A	N/A	26 (6·3)
Other nonclinical staff	N/A	N/A	166 (40·3)
**Years since professional school (Physician/APP/nurse), mean (SD)**	9·6 (9·4)	9·1 (8·0)	N/A
**Clinical work hours per week, mean (SD)**	32·5 (9·9)	35·0 (5·0)	35·4 (8·1)
**Other healthcare employment (outside COVERED sites), n (%)**	91 (10·8)	53 (12·7)	28 (6·8)
**DEMOGRAPHICS**			
**Age, mean (SD)**	37·5 (9·7)	35·7 (9·7)	42·0 (12·6)
**Gender**			
Male, n (%)	454 (53·8)	75 (18·0)	89 (21·6)
Female, n (%)	387 (45·9)	338 (81·1)	322 (78·2)
Nonbinary, n (%)	3 (0·4)	4 (1·0)	1 (0·2)
**Race**			
White, n (%)	696 (82·5)	355 (85·1)	293 (71·1)
Black, n (%)	40 (4·7)	20 (4·8)	68 (16·5)
Asian, n (%)	95 (11·3)	30 (7·2)	27 (6·6)
Another race, n (%)	13 (1·5)	12 (2·9)	24 (5·8)
**Hispanic or Latino, n (%)**	3 (0·4)	7 (1·7)	7 (1·7)

All individuals who completed weekly surveillance are included in this table, although some participated in only a portion of the 20-week period. *APP, advanced practice provider; PA, physician assistant; NP, nurse practitioner; SD, standard deviation, N/A, not applicable*
