# Supplementary material for: Emergency department personnel patient care-related COVID-19 risk
Source: PLoS One. 2022 Jul 22;17(7):e0271597. doi: 10.1371/journal.pone.0271597 (PMC9307202; doi:10.1371/journal.pone.0271597)
Supplement: S4 Table — (PDF) [file pone.0271597.s007.pdf]

S4 Table. Non-occupational exposures of participants by job category.

Characteristic	Physician/APP Participant-Weeks (n=14,775)	Nurse Participant-Weeks (n=7,596)	Non-Clinical Participant-Weeks (n=7,454)
**Co-habitants**			
No one, n (%)	2125 (14·4)	993 (13·1)	1383 (18·6)
Spouse or significant other, n (%)	11047 (74·8)	4769 (62·8)	4443 (59·6)
Roommates, n (%)	845 (5·7)	758 (10·0)	390 (5·2)
Children, n (%)	6318 (42·8)	3092 (40·7)	2936 (39·4)
Parents, grandparents, aunts, uncles, or other family, n (%)	874 (5·9)	864 (11·4)	1113 (14·9)
**Number of people in household, mean (SD)**	2.7 (1·7)	2.7 (1·8)	2.7 (3·7)
**Known contact with other friends or community members with symptoms of COVID-19, n (%)**	257 (1·6)	185 (2·4)	154 (2·1)
**Attended mass gatherings, n (%)**	1146 (7·8)	927 (12·2)	662 (8·9)
**Public transportation, n (%)**	1061 (7·2)	589 (7·8)	632 (8.5)
**Universal mask use outside work**			
Always, n (%)	10,014 (67·8)	4745 (62·5)	5840 (78·4)
Sometimes, n (%)	4372 (29·6)	2404 (31·7)	1443 (19·4)
Rarely, n (%)	252 (1·7)	323 (4·3)	121 (1·6)
Never, n (%)	64 (0·4)	89 (1·2)	30 (0·4)

SD, standard deviation
