# Supplementary material for: Emergency department personnel patient care-related COVID-19 risk
Source: PLoS One. 2022 Jul 22;17(7):e0271597. doi: 10.1371/journal.pone.0271597 (PMC9307202; doi:10.1371/journal.pone.0271597)
Supplement: S5 Table — (PDF) [file pone.0271597.s008.pdf]

S5 Table. Characteristics of participants who developed SARS-CoV-2 infection during the risk epoch.

Participant	Job type	Age category	Personal COVID-19 patients (weekly total)	Treated any COVID patients with inadequate PPE	New hospital COVID-19 admissions (weekly total)	Intubated or involved in cardiac arrest care during epoch	Other COVID-19 aerosol-generating procedure during epoch	Procedures done without CDC guideline-adherent PPE	Consistent community mask use	Attended mass gatherings or used public transit	Household COVID-19 exposure	Known community COVID-19 exposure
1	Nurse	<30	1–5	No	<40 COVID patients per week	No	No	N/A	No	Yes	No	Yes
2	Nurse	40–50	>10	Yes	<40 COVID patients per week	No	No	N/A	Yes	No	No	No
3	Attending Physician	40–50	1–5	No	<40 COVID patients per week	Yes	No	No	No	Yes	No	No
4	Nurse	40–50	>10	No	≥100 COVID patients per week	No	No	N/A	No	No	No	No
5	APP	30–40	>10	Yes	<40 COVID patients per week	No	No	N/A	No	Yes	No	No
6	Resident Physician	30–40	1–5	No	<40 COVID patients per week	Yes	No	No	No	No	No	No
7	Resident Physician	30–40	6–10	Yes	<40 COVID patients per week	No	No	N/A	Yes	Yes	No	No
8	NonClinical Staff	40–50	0	N/A	<40 COVID patients per week	N/A	N/A	N/A	Yes	Yes	No	Yes
9	Attending Physician	40–50	6–10	Yes	<40 COVID patients per week	Yes	Yes	Yes	Yes	No	No	Yes
10	Nurse	30–40	1–5	Yes	40–99 COVID patients per week	Yes	Yes	No	Yes	Yes	No	No
11	Nurse	<30	6–10	No	<40 COVID patients per week	No	No	N/A	No	Yes	Yes	Yes
12	Non-Clinical Staff	40–50	0	N/A	<40 COVID patients per week	N/A	N/A	N/A	Yes	No	No	No
13	Non-Clinical Staff	30–40	0	N/A	<40 COVID patients per week	N/A	N/A	N/A	No	No	No	Yes
14	Resident	<30	1–5	Yes	<40 COVID patients per week	Yes	No	Yes	Yes	Yes	No	No
15	Nurse	30–40	6–10	No	<40 COVID	Yes	No	No	No	Yes	No	Yes

					patients per week							
16	Attending Physician	30–40	1–5	Yes	40–99 COVID patients per week	No	No	N/A	No	No	No	Yes
17	NonClinical Staff	>50	0	N/A	<40 COVID patients per week	N/A	N/A	N/A	Yes	Yes	No	No
18	Nurse	30–40	6–10	No	<40 COVID patients per week	No	Yes	No	Yes	No	No	No
19	Nurse	>50	1–5	No	<40 COVID patients per week	Yes	Yes	No	Yes	No	No	No
20	Attending Physician	30–40	>10	No	≥100 COVID patients per week	Yes	Yes	No	No	No	No	Yes
21	Attending Physician	40–50	1–5	Yes	<40 COVID patients per week	No	Yes	Yes	Yes	No	No	No
22	Non-Clinical Staff	30–40	0	N/A	<40 COVID patients per week	N/A	N/A	N/A	Yes	No	No	No
23	Nurse	<30	6–10	Yes	<40 COVID patients per week	Yes	Yes	No	Yes	Yes	No	No
24	Non-Clinical Staff	40–50	0	N/A	<40 COVID patients per week	N/A	N/A	N/A	No	No	No	No
25	Nurse	30–40	6–10	Yes	<40 COVID patients per week	Yes	No	No	Yes	Yes	No	Yes
26	APP	30–40	6–10	Yes	≥100 COVID patients per week	No	No	N/A	Yes	No	No	No
27	Resident Physician	<30	1–5	Yes	<40 COVID patients per week	Yes	Yes	Yes	No	Yes	No	Yes
28	Attending Physician	30–40	>10	No	<40 COVID patients per week	No	Yes	No	Yes	No	No	No
29	Attending Physician	>50	6–10	Yes	≥100 COVID patients per week	No	No	N/A	Yes	No	No	No
30	Nurse	30–40	1–5	Yes	<40 COVID patients per week	No	No	N/A	No	Yes	Yes	No
31	Nurse	40–50	>10	Yes	<40 COVID patients per week	No	No	N/A	Yes	No	No	No
32	Non-Clinical Staff	>50	0	N/A	<40 COVID patients per week	N/A	N/A	N/A	Yes	No	No	No
33	Nurse	40–50	1–5	No	40–99 COVID	No	No	N/A	Yes	No	No	No

					patients per week							
34	Attending Physician	40–50	>10	Yes	≥100 COVID patients per week	Yes	Yes	Yes	No	Yes	No	No
35	Attending Physician	30–40	6–10	Yes	40–99 COVID patients per week	Yes	No	Yes	Yes	No	No	No
36	Nurse	<30	>10	Yes	≥100 COVID patients per week	Yes	No	Yes	Yes	No	No	No
37	Nurse	30–40	6–10	No	<40 COVID patients per week	Yes	Yes	No	Yes	No	No	No
38	Nurse	30–40	1–5	Yes	<40 COVID patients per week	No	Yes	Yes	Yes	No	No	No
39	Nurse	<30	>10	Yes	≥100 COVID patients per week	No	No	N/A	No	No	Yes	No
40	Nurse	<30	1–5	Yes	40–99 COVID patients per week	Yes	No	Yes	Yes	Yes	No	No
41	Resident Physician	<30	1–5	No	<40 COVID patients per week	Yes	No	No	No	Yes	Yes	Yes
42	APP	30–40	1–5	Yes	<40 COVID patients per week	No	No	N/A	Yes	Yes	No	No
43	Nurse	<30	6–10	Yes	≥100 COVID patients per week	No	Yes	Yes	No	No	No	No
44	Non-Clinical Staff	<30	0	N/A	40–99 COVID patients per week	N/A	N/A	N/A	Yes	Yes	No	No
45	Nurse	<30	6–10	No	<40 COVID patients per week	No	No	N/A	No	Yes	No	No
46	Non-Clinical Staff	30–40	0	N/A	40–99 COVID patients per week	N/A	N/A	N/A	No	Yes	No	No
47	Non-Clinical Staff	30–40	0	N/A	<40 COVID patients per week	N/A	N/A	N/A	No	No	Yes	No
48	Resident Physician	<30	>10	Yes	<40 COVID patients per week	Yes	No	No	No	No	Yes	No
49	Attending Physician	30–40	>10	Yes	40–99 COVID patients per week	No	No	N/A	No	No	No	No
50	Resident	<30	>10	Yes	40–99	Yes	Yes	Yes	Yes	Yes	Yes	Yes

	Physician				COVID patients per week							
51	Non-Clinical Staff	<30	0	N/A	40–99 COVID patients per week	N/A	N/A	N/A	Yes	No	No	No
52	Nurse	<30	6–10	Yes	40–99 COVID patients per week	Yes	No	Yes	Yes	Yes	No	Yes
53	Nurse	<30	1–5	Yes	40–99 COVID patients per week	Yes	Yes	Yes	No	Yes	No	No
54	Non-Clinical Staff	<30	0	N/A	40–99 COVID patients per week	N/A	N/A	N/A	Yes	Yes	No	Yes
55	Resident Physician	<30	0	No	<40 COVID patients per week	No	No	N/A	Yes	No	No	No
56	Attending Physician	40–50	>10	No	40–99 COVID patients per week	Yes	Yes	Yes	Yes	No	No	No
57	Non-Clinical Staff	<30	0	N/A	40–99 COVID patients per week	N/A	N/A	N/A	Yes	No	No	No
58	Non-Clinical Staff	40–50	0	N/A	40–99 COVID patients per week	N/A	N/A	N/A	No	No	No	No
59	Resident Physician	30–40	1–5	Yes	<40 COVID patients per week	Yes	Yes	No	Yes	No	No	No
60	Resident Physician	<30	6–10	No	≥100 COVID patients per week	Yes	Yes	No	Yes	No	No	No
61	Resident Physician	<30	6–10	Yes	40–99 COVID patients per week	Yes	Yes	Yes	No	No	No	No
62	Nurse	40–50	>10	No	<40 COVID patients per week	Yes	No	No	No	No	No	No
63	Resident Physician	<30	>10	Yes	≥100 COVID patients per week	Yes	No	No	Yes	No	No	No
64	Resident Physician	<30	>10	Yes	<40 COVID patients per week	Yes	Yes	No	No	No	No	Yes
65	Nurse	<30	1–5	Yes	<40 COVID patients per week	No	No	N/A	No	No	No	No
66	Resident	<30	6–10	Yes	40–99	Yes	No	Yes	No	No	No	No

	Physician				COVID patients per week							
67	Non-Clinical Staff	>50	0	N/A	<40 COVID patients per week	N/A	N/A	N/A	No	Yes	No	No
68	Attending Physician	30–40	1–5	Yes	<40 COVID patients per week	Yes	Yes	No	No	No	No	No
69	Nurse	30–40	1–5	Yes	<40 COVID patients per week	No	No	N/A	No	Yes	No	No
70	Nurse	<30	6–10	No	<40 COVID patients per week	No	No	N/A	No	Yes	No	No
71	Resident Physician	<30	>10	No	≥100 COVID patients per week	Yes	No	No	Yes	No	No	No
72	Nurse	<30	>10	Yes	<40 COVID patients per week	Yes	Yes	Yes	No	Yes	Yes	No
73	APP	>50	>10	No	≥100 COVID patients per week	No	No	N/A	Yes	No	No	No
74	Nurse	30–40	1–5	Yes	<40 COVID patients per week	No	No	N/A	No	No	No	No
75	Nurse	30–40	6–10	Yes	<40 COVID patients per week	Yes	Yes	No	No	No	No	No
