# Supplementary material for: Emergency department personnel patient care-related COVID-19 risk
Source: PLoS One. 2022 Jul 22;17(7):e0271597. doi: 10.1371/journal.pone.0271597 (PMC9307202; doi:10.1371/journal.pone.0271597)
Supplement: S6 Table — (PDF) [file pone.0271597.s009.pdf]

S6 Table. Multivariable Regression Models for Risk Factor Analysis.

Factor	Unadjusted OR (95% CI)	Model 1 aOR (95% CI)	Model 2 aOR (95% CI)
Household exposure vs None	28·23 (11·57–64·09)	16·34 (5.79–46·09)	16·62 (6·09–45·35)
Hospital COVID-19 volume (>100 vs <=100 patients/week)	5·08 (2·69–9·57)	3·28 (1·68–6·40)	3·21 (1·66–6·23)
Community COVID-19 cumulative incidence (15+ vs. <15 per 100,000 population per week)	4·34 (2·73–6·90)	3·21 (1·95–5·29)	3·03 (1·85–4·96)
Community COVID-19 exposure vs None	4·02 (2·25–7·16)	2·38 (1·24–4·54)	2·34 (1·23–4·45)
Physician vs. non-clinical	1·03 (0·55–1·91)	1·07 (0·56–2·03)	
Nurse vs. Non-Clinical	1·92 (1·03–3·57)	1·91 (0·99–3·68)	
COVID-19 intubating physician vs.non-clinical	3·65 (1·39–9·58)		2·75 (1·08–6·99)
Non-COVID-19 intubating physician vs. non-clinical	1·32 (0·63–2·76)		1·27 (0·59–2·73)
Non-intubating physician vs. non-clinical	0·61 (0·28–1·34)		0·70 (0·31–1·56)
Intubating nurse vs. non-clinical	1·99 (0·76–5·12)		2·01 (0·93–4·32)
Non-intubating nurse vs. non-clinical	1·86 (0·92–3·77)		1·83 (0·87–3·84)
