# Supplementary material for: Emergency department personnel patient care-related COVID-19 risk
Source: PLoS One. 2022 Jul 22;17(7):e0271597. doi: 10.1371/journal.pone.0271597 (PMC9307202; doi:10.1371/journal.pone.0271597)
Supplement: S7 Table — (PDF) [file pone.0271597.s010.pdf]

S7 Table. Recursive Partitioning Analysis

Level	Factor	Percentage of participants with factor remaining among those who developed COVID-19	Percentage of participants with factor among those who did not develop COVID-19
1	Weekly hospital COVID-19 volume (>100 patients per week)	26·7%	6·3%
2	Community COVID-19 Cumulative incidence (≥15 cases per 100,000 population)	52·7%	23.0%
3	Face mask use in community ("Always")	53·8%	66.9%

The recursive partitioning analysis is an iterative analysis in which each risk factor that most strongly predicts COVID-19 infections is identified, then positive cases are removed from the overall cohort. This table shows each risk factor and the strength of the association at each level of partitioning.
