# Supplementary material for: Emergency department personnel patient care-related COVID-19 risk
Source: PLoS One. 2022 Jul 22;17(7):e0271597. doi: 10.1371/journal.pone.0271597 (PMC9307202; doi:10.1371/journal.pone.0271597)
Supplement: S1 File — (PDF) [file pone.0271597.s011.pdf]

S1 File. COVID Evaluation of Risk for Emergency Departments (COVERED) Project: Project Protocol

Co-Principal Investigators: Nicholas Mohr, MD, MS & David Talan, MD

Funding: This project was conducted with funding from the Centers for Disease Control and Prevention (CDC), Award #U01CK000480-05 and from the University of Iowa Institute for Clinical and Translational Sciences (NIH CTSA award UL1TR002537).

Version number: 5.0

Date: September 16, 2020

Table of Contents

1.0 Background 2
2.0 Project Objectives 2
3.0 Project Design and methods 3
  3.1 Inclusion criteria 4
  3.2 Exclusion criteria 4
  3.3 Recruitment and enrollment 4
  3.4 Project data collection 5
    3.4.1 Baseline and weekly surveys 5
    3.4.2 Capturing aerosol-generating procedures 5
    3.4.3 Site weekly facility surveys 6
    3.4.4 Extended follow-up surveys 6
    3.4.5 Blood and nasal swab collection 6
  3.5 Participant compensation 6
  3.6 Withdrawals 7
  3.7 Central laboratory testing 7
    3.7.1 Interpretation of laboratory testing results 8
4.0 Data management and analysis 8
  4.1 Data management 8
  4.2 Statistical Analysis 9
    4.2.1 Sample Size Estimation 9
    4.2.2 Analysis strategy 10
6.0 Protection of Human Subjects 11
  6.1. Informed Consent 12
7.0 References 12

1.0 Background

Rapidly spreading infectious diseases with community transmission put healthcare personnel (HCP) at risk because patients at the early stage of illness may be difficult to identify, personal protective equipment may not be readily available, and specific modes of transmission may not be well understood. On March 11, 2020, the World Health Organization (WHO) declared COVID-19 a worldwide pandemic, involving at least 114 countries resulting in more than 4,000 deaths. Within one month, the worldwide death toll had surpassed 108,000.

In health systems affected early in the pandemic, healthcare personnel were a particularly high-risk group for acquiring the disease, presumably due to workplace exposure. Over one-third of those infected in a COVID-19 case-series in Wuhan, China and 9% of the total cases in Italy were HCP.[1, 2] These findings parallel data from the 2003 SARS-1 and MERS outbreaks, both of which documented HCP transmission.[3,4]

SARS-CoV-2, the causative agent in COVID-19 infection, is thought to spread primarily through close personal contact and respiratory droplets. In the setting of emergency department (ED) care, additional transmission risk exists due to lack of knowledge of which patients are COVID-infected, unexpected arrival of critically ill patients, and the need to regularly perform life-saving procedures such as endotracheal intubation. Endotracheal intubation was associated with substantially increased risk for infection of HCW during the SARS epidemic, presumably due to aerosolization of virus, and is considered a high-risk event for transmission of COVID-19. Intubation alternatives such as noninvasive positive pressure ventilation and high-flow nasal cannula have also raised safety concerns. As the COVID-19 pandemic spreads throughout the U.S., EDs have been stretched to capacity, and the number of COVID-19 patients with acute respiratory failure requiring endotracheal intubation has been increasing exponentially.

Emergency providers are critically needed to respond to the COVID-19 pandemic, so it is essential to maintain their health and ability to work. The goals of the **COVERED** project are to assess the burden and associated risk factors among HCP for COVID-19 acquisition and identify means to prevent transmission to HCPs in emergency care, with the overall objective to inform response activities for maintaining the emergency care community during the COVID-19 and future pandemics.

2.0 Project Objectives

The objectives of this project are to:

1) Estimate the attributable risk of occupational acquisition of COVID-19 infection during emergency care for SARS-CoV-2-positive patients;
    a. among physicians who perform endotracheal intubation in the ED and other aerosol-generating procedures,
    b. among physicians performing non-intubation care in the ED,

    c. among ED nurses (stratified by involvement in aerosol-generating procedures), and
    d. among family members of ED HCPs (relative to population prevalence).
2) Identify patient-, provider-, facility- and procedure-based risk factors associated with SARS-CoV-2 transmission during endotracheal intubation;
3) Estimate the overall burden of COVID-19 infections occurring among ED HCPs in a cohort of EDs (measured from facility-level data in infections-per-hour of patient care stratified by physician, nurse, and non-clinical staff);
4) Determine the prevalence of asymptomatic and symptomatic COVID-19 illness and viral shedding in ED HCPs; and
5) Describe current strategies of airway management and personal protective equipment (PPE) use in COVID-19 patients during the 2020 pandemic.
6) Describe return to work practices among HCPs with COVID-19 infection or seroconversion during the study period.

3.0 Project Design and methods

This project is a multicenter observational enrolled-cohort surveillance conducted in high-volume centers treating COVID-19 patients over a 20-week period. Sites will be recruited from the following two national ED-based research networks and additional high-volume sites with appropriate infrastructure and active local COVID-19 activity:

1) *EMERGE*ncy IDNet[6] – This CDC-funded 12-site ED-based emerging infectious disease network was created for surveillance and research of emerging infectious diseases (PI: David Talan, MD); and
2) **National Emergency Airway Registry (**NEAR**)** – This 26-site network is the largest ED-based research network focused on a multicenter observational airway management studies (PI: Calvin Brown, MD).

Participating sites will enroll a population of HCPs (physicians, nurses, and non-clinical staff) working in EDs likely to care for patients with COVID-19. Providers will participate in voluntary surveillance activities with prospectively collected data on endotracheal intubations and other aerosol-generating procedures (patient- and procedure-level data), other unprotected high-risk exposure events, SARS-CoV-2 serology tests, and viral PCR from self-administered nasal swabs. Non-clinical staff will serve as the control group to capture the risk of community transmission and transmission between HCPs (separate from patient exposure) in the work environment. These HCPs will be followed for an estimated 5 months during the study period to evaluate serologic and PCR evidence of SARS-CoV-2 exposure and/or clinical symptoms of COVID-19 infection. Local COVID-19 activity will be monitored using standard public health reporting (from reporting collated by the CDC Science Office) and facility-specific admissions (collected from weekly surveys at all sites).

3.1 Inclusion criteria

Three groups of ED HCPs will be recruited:

1) physicians
2) nurses
3) non-clinical ED staff who have no clinical patient contact with COVID-19 patients.

For the purposes of calculating attributable risk, four cohorts will be defined. The following cohorts are at risk of COVID-19 acquisition from other HCPs and community contacts.

1) **Aerosol-Exposed Physician Cohort** – Emergency physicians will be included in the cohort if they have intubated COVID-19 patients (defined as a confirmed positive SARS-CoV-2 PCR test either within the 14 days before or two days after intubation) during the study period. Aerosol-exposed physicians typically are senior residents and attending emergency physicians;
2) **Non-aerosol-Exposed Physician Cohort** – Emergency physicians who have not intubated COVID-19 patients either because they did not intubate or because none of their patients were found to be COVID-infected during the study period. For the purpose of analysis, physicians may cross over between groups based on the patients that they treat during the surveillance period. This group would typically include junior residents and some attending emergency physicians.
3) **Nurse Cohort** – ED nurses, who are treating patients in the ED, will be included in this cohort. Specific information on exposures, e.g., participating in intubation, management of critically ill COVID patients, will be collected to measure risk.
4) **Non-Clinical Exposure Cohort** - Surveillance site ED employees who do not participate in endotracheal intubation procedures or direct patient care will be included. Non-clinical exposure patients include unit clerks, social workers, scribes, and case managers.

3.2 Exclusion criteria

1) HCPs unable to complete surveillance follow-up visits (e.g., temporary HCPs; Note: an exception will be made for graduating senior residents who perform most of or all of the intubations at the site if they are expected to be able to complete at least two consecutive blood draws/swabs);
2) HCPs who have previously been infected with COVID-19; and
3) HCPs who decline to provide informed consent.

3.3 Recruitment and enrollment

Site teams will recruit HCP by announcing the project at their sites through email, presentations at meetings, and/or in person. They will compile a recruitment list of potential participants who would most likely meet the above inclusion criteria. This list should include a minimum of 20 potential recruits in each of the four cohorts and if possible, additional HCPs for each cohort in

case a potential recruit does not qualify or is enrolled and later withdrawn. When the site is released to start enrollment by the main coordinating site, they will invite HCP by emailing them a link to the REDCap database, where they will complete a screening form to confirm eligibility.

If eligible, HCP will be asked to provide electronic informed consent, and if they consent to participate, they will be enrolled in the project and assigned a project ID. Project data collection will start immediately.

3.4 Project data collection

All participants will complete weekly surveys and regular blood draws for testing for serum IgG and self-administered nasal swabs for PCR to determine COVID-19 infection (see Section 3.4.5). In addition, participants who cared for or supervised the care of (i.e., was within three feet of) a patient who required intubation or who was in cardiac arrest, will complete a form for each patient encounter. HCPs should follow their local institutional guidance on the use of PPE for COVID-19 protection.

3.4.1 Baseline and weekly surveys

The participant will complete a baseline survey at enrollment and surveys every week through 20 weeks. The baseline survey will collect personal and emergency contact information, demographics, COVID testing (outside of project), COVID exposures (at work and at home), COVID-19-related stress and anxiety, current living situation and medical history. The weekly survey will ask about COVID symptoms, COVID diagnosis (including medications, hospitalizations, and treatment, if applicable), COVID exposures, use of PPE, changes in PPE usage in the last week, including PPE protocols and shortages at their institutions, changes to living situation and contact information. All surveys can be completed outside clinical service, so participation should not interfere with clinical care.

3.4.2 Capturing aerosol-generating procedures

For every intubation or cardiac pulmonary resuscitation (CPR) performed by the participant in the ED, a case report form will be completed (within 24 hours) to collect patient-specific and procedure-specific data elements. Participants who are supervising residents performing these procedures and are within three feet of the patient will also be asked to complete a case report form. Data on cardiac arrest patients will also be captured on the same form (even if patients are intubated prior to ED arrival). Site coordinators will monitor all intubations and cardiac arrests performed in the ED and will remind participants to complete the form within 24 hours of these events for specific patient or event-related data. Site coordinators will also review medical records of all patients requiring intubation or CPR to note COVID infection status. On weekly their surveys, nurses will be asked if they assisted or were involved in intubation and CPR events, as well.

3.4.3 Site weekly facility surveys

Site coordinators will complete a weekly survey to report facility-level variables, including PPE supplies, infection control variables, COVID rates among patients, and overall COVID rates among HCP, and total hours worked for each of the employee groups to calculate a rate of infections/1000 hours worked.

3.4.4 Extended follow-up surveys

Approximately three months after the initial 20-week follow-up period, participants will be asked to complete an extended follow-up survey to obtain information about subsequent COVID infection, changes in work practices and PPE use (at home and at work), anxiety, burnout, whether they were vaccinated, and reasons for vaccine acceptance or refusal. Site coordinators will also complete a facility survey to record information about subsequent COVID-19 infections among ED staff.

3.4.5 Blood and nasal swab collection

Blood and nasal swab collection will occur every two weeks for four weeks and then every four weeks for a total of 20 weeks as outlined in the table below to determine baseline and subsequent COVID-19 infection status.

Week 0 Baseline Survey completion (+/- 2 days)	Week 2 Target Date (+/- 2 days)	Week 4 Target Date (+/- 4 days)	Week 8 Target Date (+/- 4 days)	Week 12 Target Date (+/- 4 days)	Week 16 Target Date (+/- 4 days)	Week 20 Target Date (+/- 4 days)
Day 1	Day 14	Day 28	Day 56	Day 84	Day 112	Day 140

Blood draws will be performed by phlebotomists at local sites and self-administered nasal swabs will be collected at the time of each blood draw for PCR testing. Site coordinators will instruct participants on how to collect self-administered nasal swabs.

3.5 Participant compensation

All participants will be reimbursed for participation including completing multiple surveys and laboratory blood draws and nasal swabs. Shortly after enrollment, participants will receive a Visa debit card by mail. The Data Coordinating Center will manage reimbursements to participants for each activity (e.g., for each blood draw, nasal swab, survey) completed by loading additional value on the debit card weekly.

3.6 Withdrawals

Reasons for withdrawals include:

1) participant's initial blood (serology) draw or nasal swab (PCR) is positive for COVID infection;
2) participant chooses to not continue to participate or has to move on to another job; or
3) participant is noncompliant with returning for their blood draws/swab collections and/or completing their surveys; or
4) participant has a medical condition or issue with providing blood for testing such that the phlebotomist is not able to consistently (i.e., two or more consecutive blood draws) draw enough blood from them required for testing.

3.7 Central laboratory testing

All samples will be stored at sites and shipped at least weekly overnight to the central laboratory (ARUP laboratories, Salt Lake City, UT). Specimens will be deidentified prior to shipping and will be accompanied by standard documentation and labeling. Detailed procedures for sample handling, storage, and shipment will be sent to sites prior to project launch. Results will be reported in two to six days from receipt of specimens back to the Data Coordinating Center (DCC), who will distribute test results to all participants (approximately six to 12 days after sample collection).

The blood samples will be tested for antibodies to SARS-CoV-2 using the Abbott anti-SARS-CoV-2 IgG test. The results of this test are reported as "negative," "indeterminate," and "positive." This test received an Emergency Use Authorization (EUA) from the FDA, but no current anti-SARS-CoV-2 antibody tests have received FDA approval. Samples that yield a positive result on the Abbott test will be confirmed using another antibody assay, the EUROIMMUN test. This test provides both a qualitative interpretation and a quantitative interpretation. The ratio-based quantitative result is classified as follows:

- Ratio $< 0{\cdot}8$ = Negative
- $0{\cdot}8 \leq$ Ratio $<1{\cdot}1$ = Borderline/Indeterminate
- Ratio $\geq 1{\cdot}1$ = Positive

The nasal swab will undergo PCR testing for SARS-CoV-2 virus particles. Self-inserted nasal swabs are less invasive than clinician-inserted nasopharyngeal swabs, and they are currently thought to have similar sensitivity. This test has received an Emergency Use Authorization (EUA) from the FDA, but no current SARS-CoV-2 viral tests have received FDA approval.

3.7.1 Interpretation of laboratory testing results

At baseline, participants with positive results on either the Abbott test or the nasal swab PCR test will be considered as positive for SARS-CoV-2 and withdrawn from the study. Participants with positive results at baseline on the Abbott test and negative or indeterminate on the EUROIMMUN test will be withdrawn from the study, since the tests might have different performance characteristics in these individuals.

After baseline, results of the laboratory tests will be interpreted as follows:

- Participants with positive results on both the Abbott test and the EUROIMMUN test will be considered as positive for SARS-CoV-2.
- Participants with positive results on the Abbott test and negative or indeterminate on the EUROIMMUN test will be considered negative for SARS-CoV-2.
- Participants with positive nasal swab RT-PCR results will be considered as positive for SARS-CoV-2.

Any participant who is found to seroconvert or has a positive nasal swab PCR will be notified immediately by an appointed site team member and advised to get evaluation by local employee health and public health authorities for screening and return to work determination according to current public health guidelines. Participants who contract COVID-19 during the project period will continue to complete weekly surveys but will not continue with blood draws and nasal swabs.

4.0 Data management and analysis

4.1 Data management

All data will be managed centrally at the Data Coordinating Center (DCC) located at the University of Iowa. All forms will be completed by participants and site teams electronically using REDCap.[6]

The following data will be maintained on the REDCap database:
1) Informed consent documents,
2) Participant survey data
3) Weekly facility forms completed by the site coordinators,
4) Intubation and CPR event forms completed by participants,
5) Patient information forms completed by the site teams for each intubation and CPR event, and
6) Laboratory testing results from the central laboratory

The DCC will set up reports within REDCap to track data quality, collection of specimens, and reporting of laboratory results in a timely fashion. They will also set up email and text message prompts for participants to complete their surveys at the required timepoints. The database will also contain logic checks and require responses for all survey questions to ensure the data are entered accurately and completely.

4.2 Statistical Analysis

4.2.1 Sample Size Estimation

To estimate the power of our surveillance design, we simulated a series of data sets based on the following assumptions:

Baseline population infection risk during study period=1% (estimated at 0·6% based on CDC modeling)
Attributable risk of 5% (based on MERS transmission with maximal precautions[37])
Sites=20 hospitals
Physician participants at each hospital=30
Standard deviation for variability in transmission risk between hospitals=3%
Standard deviation for variability in transmission risk between individuals within a hospital=3%
Probability of intubating COVID-19 patient during 2-week period=20%
Alpha=5%

Table. Assumptions for power calculation simulation.

These assumptions were built into a simulation replicated 100 times to estimate the power to detect the 5% estimated risk difference. According to data on HCP infections in long-term care facilities, an estimated 20% infection proportion was observed.

Sensitivity analysis was used to measure the impact of changes in the assumption values, and this analysis is reflected in Figure 1.

Separate analysis was conducted by Bill Mower (UCLA) using an explicit estimation of correlation between intubators and by James Baggs (CDC) using survival analysis. Both cohorts estimated similar effect size with slightly more power using survival analysis. Because the simulation uses a clustered analysis with less power than the planned final analysis, we conservatively used these estimates.

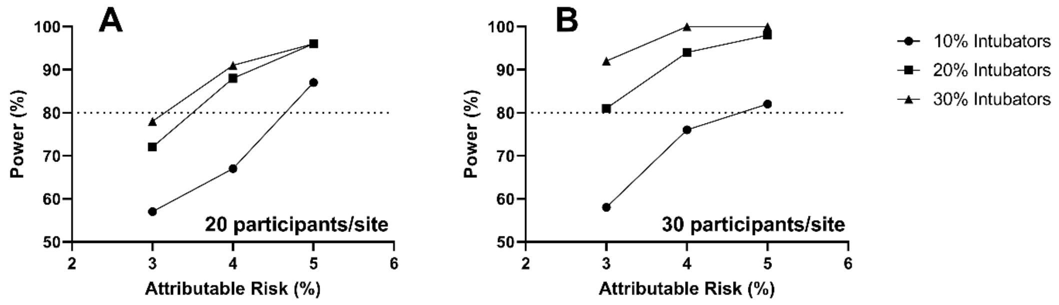

Figure 1. Sensitivity analysis for sample size estimation. Panel A shows the power analysis for 20 physicians/site and **Panel B** shows the power analysis for 30 physicians/site. The minimum detectable attributable risk is related to power. The 3 lines represent the proportion of total 2-week periods at a site in which a given study participant performs an aerosol-generating procedure.

Assuming 25% loss-to-follow-up, we plan to enroll 40 physicians, 20 nurses, and 20 nonclinical staff at each of 20 sites (1600 participants). All secondary analyses will have similar power. This simulation suggests that our **power is 94%** to detect a difference in COVID-19 acquisition of 4%.

4.2.2 Analysis strategy

Two strategies of modeling will be used to estimate the effect of endotracheal intubation on seroconversion risk. The **event-level analysis** will use a data set with one record for every COVID-19 intubation, and the outcome will be the seroconversion between 1 and 14 days. The **epoch-level analysis** will use a longitudinal data set with time-dependent covariates with one record for each time epoch, and the exposures for the prior 2 weeks will be independent variables in the model. Epoch-level analysis will include physician, nurse, and non-clinical exposure cohorts. For each epoch, physicians will be classified as aerosol-exposed or non-aerosol exposed, and aerosol-exposed physicians will record a count of aerosol-generating procedures for dose-response analysis. All participating nurses will be included, and weekly data on their exposure to aerosol-generating procedures will be collected as an exposure. All models will be generated using a hierarchical structure (provider and facility) with Generalized Linear Mixed Model to account for clustering, and analysis for attributable risk will be conducted using survival analysis with time-dependent covariates.

4.2.2.1 Estimate attributable risk - Attributable risk is calculated as the difference in disease incidence in the exposed and the unexposed. We will build an extended Cox proportional hazards model (survival analysis) with time-dependent covariates to account for baseline risk, evolving practice over the project period, and provider-level variables. We will include a 3-level categorical variable for provider type (Physician, Nurse, and Non-Clinical Staff), and we will include a separate variable for the count of aerosol-generating procedures (within each 2-week period, for measure of dose-response relationship). The variables of interest will be the provider-type variable and the count of aerosol-generating procedures during the lagging 2 weeks, which will provide the hazard ratio for the risk of clinical care vs. non-clinical exposure

(reference group) and the risk of endotracheal intubation (and other aerosol-generating procedures) vs. clinical care alone. From this analysis, we will calculate an adjusted attributable risk.

4.2.2.2 Identify patient-, provider-, and procedure-based risk factors associated with SARS-CoV-2 transmission - Specific potentially modifiable procedure-related factors associated with SARS-CoV-2 transmission will be identified using the event-level analysis. For each case, the outcome will be whether seroconversion occurred within the subsequent two weeks. Patient-, provider-, facility-, and procedure-level factors will be included as predictors in the model to determine the relative contribution of these factors to the probability of seroconversion. We will use a principal components analysis to identify the factors most strongly associated with that risk.

4.2.2.3 Identify the extent of subclinical COVID-19 illness in HCPs – From the weekly symptom surveys, we will identify the proportion of HCPs who developed COVID-19 without symptoms (based on seroconversion without symptoms). This will be reported as a simple prevalence.

4.2.24 Describe current strategies of airway management and PPE use in COVID-19 patients during the 2020 pandemic – Report descriptive statistics (means, medians) for how airway management is performed in COVID-19 patients at various institutions during the 2020 epidemic.

4.2.5 Assess overall risk of HCP acquisition – Weekly reports of the number of staff (by provider type) who have been newly infected with COVID-19, along with number of hours of coverage by provider type. This will allow an incidence per 100/hours worked to be calculated across all centers. This analysis will not be restricted to the enrolled cohort—it will be across all employees in participating EDs.

4.2.6 Risk of transmission to HCP family members – Weekly reports will collect data on COVID-19 infections among family members. We will report descriptive analysis for family members, along with the timing of family member COVID-19 infections (e.g., before or after HCP seroconversion).

6.0 Protection of Human Subjects

This project has been determined by the Centers for Disease Control and Prevention (CDC), the University of Iowa IRB, and the Olive View-UCLA IRB not to be human subjects research because it constitutes public health surveillance activities. All participant data will be kept confidential using password-protected research servers accessible only to the study team. No testing results or survey data will be shared with employers or public health authorities. Data from individual sites will not be identified, and all data will be reported in aggregate. Each site IRB will make an independent determination about whether study activities constitute human subjects research, and data use agreements will be executed with the DCC as required by individual sites.

6.1. **Informed Consent**

Participation in the surveillance is strictly voluntary. All HCPs who are eligible for participation will provide electronic informed consent to participate. This activity is consistent with non-research public health surveillance (by determination of the CDC and IRBs at the University of Iowa and Olive View-UCLA).

All participant and patient protected health information will be maintained in a confidential, password-protected secure electronic form. The risks to participant HCPs are minimal and include 1) the time required to complete surveys, 2) pain and discomfort associated with needlesticks and self-administered nasal swabs for serial COVID-19 serology testing and screening for viral shedding, and 3) inadvertent release of protected health care information, which will be kept secure and will only be reported in aggregate.

The surveillance will require site coordinators to collect surveys from participants (electronically) and phlebotomists to collect blood samples. The surveillance poses no more than minimal risk of COVID-19 exposure because all surveys will be conducted remotely, and blood will only be drawn from patients who are asymptomatic by trained phlebotomists. Any participant diagnosed with active COVID-19 infection will not have any more in-person visits with project staff or blood or nasal specimen collection (although ongoing data collection will occur electronically).

7.0 References

1. Wang D, Hu B, Hu C, et al. Clinical Characteristics of 138 Hospitalized Patients With 2019 Novel Coronavirus–Infected Pneumonia in Wuhan, China. *JAMA* 2020;323:1061-9.
2. Borghese L, Donato VD, Ruotolo N, Fiegener J. Nearly 1 in 10 of Italy's infected are health care workers. CNN 2020 22 Mar.
3. Ofner-Agostini M, Gravel D, McDonald LC, et al. Cluster of cases of severe acute respiratory syndrome among Toronto healthcare workers after implementation of infection control precautions: a case series. *Infect Control Hosp Epidemiol* 2006;27:473-8.
4. Alraddadi BM, Watson JT, Almarashi A, et al. Risk Factors for Primary Middle East Respiratory Syndrome Coronavirus Illness in Humans, Saudi Arabia, 2014. *Emerg Infect Dis* 2016;22:49-55.
5. Talan DA, Moran GJ, Mower WR, et al. *EMERGE*ncy ID NET: An emergency department-based emerging infections sentinel network. *Clin Infect Dis.* 1999;28:401-2.
6. Paul A. Harris, Robert Taylor, Robert Thielke, Jonathon Payne, Nathaniel Gonzalez, Jose G. Conde, Research electronic data capture (REDCap) - A metadata-driven methodology and workflow process for providing translational research informatics support, *J Biomed Inform* 2009 Apr;42(2):377-81.
