# Supplementary material for: Emergency department personnel patient care-related COVID-19 risk
Source: PLoS One. 2022 Jul 22;17(7):e0271597. doi: 10.1371/journal.pone.0271597 (PMC9307202; doi:10.1371/journal.pone.0271597)
Supplement: S2 File — (PDF) [file pone.0271597.s012.pdf]

S2 File. Supplemental Statistical Procedures

We used the generalized linear mixed model (GLMM) framework to assess risk of COVID-19 infection for individual, facility, and community fixed-effect factors within each HCP epoch. All epoch-level models included nested random effects for participant within facility, adjusting for multiple epoch observations per HCP and within-site correlation. We specified a binary outcome distribution and logit link function, providing odds ratio point and interval estimates.

We fitted univariate models to assess the risk of COVID-19 infection for each of the individual fixed-effect factors. Next, we constructed multivariable models to address confounding and obtain more accurate estimates for statistically significant risk factors. A backwards selection procedure started by fitting the saturated model (containing all predictors) and iteratively removing the least important risk factor as determined by the Akaike information criterion (AIC).[1,2] This process continued until no improvements to the predictor set could be made through variable removal. Epoch duration and interaction terms between each factor and the epoch duration were tested but were found not to improve model fit. We calculated adjusted estimates and confidence intervals for absolute risk and attributable risk fraction for each job category with and without accounting for intubation.

We calculated attributable risk fraction (AF) using adjusted odds ratio estimates from regression modeling [$AF = p_e(OR-1)/(1+ p_e(OR-1))$, where p_e is the exposure prevalence], and we interpreted AF as the fraction of total COVID-19 infections attributable to a particular risk factor.[3,4] We calculated adjusted absolute risk of infection within subgroups using regression

analysis as described, then we multiplied the resulting point estimates (in person-epochs) by the mean epoch duration (weeks/epoch) to estimate the infection rate (in person-weeks).

We additionally employed a modified χ^2 recursive partitioning analysis to evaluate the association between community, hospital, and individual factors and COVID-19 infection among HCP.[5] At each partitioning level, we identified the factor that demonstrated the largest χ^2 value between HCP who had acquired COVID-19 infections and those who had not. We had designated a priori that partitioning would continue until one of the following end-points was reached: 1) all criteria were exhausted (i.e., all factors exhibited discriminating ability, but in combination did not account for all HCP COVID-19 infections); 2) all COVID-19 infected HCP were exhausted (i.e. the partitioning identified a limited set of factors that adequately characterized all COVID-19 infected HCP); or 3) remaining factors no longer distinguished additional cases. We designated the factors that exhibited the greatest discriminating capacity at each stage in partitioning process as our high-risk criteria.

References

1. Akaike H. A new look at the statistical model identification. *IEEE Transactions on Automatic Control.* 1974;19(6):716-723.
2. Akaike H. Information theory and an extension of the maximum likelihood principle. In: Petrov N, Csaki F, eds. *2nd International Symposium on Information Theory* Akademia Kiado, Budapest1973:267-281.
3. Ramakrishnan V, Thacker LR. Population Attributable Fraction as a Measure of Heritability in Dichotomous Twin Data. *Commun Stat Simul Comput.* 2012;41(3).
4. Cox C, Li X. Model-based estimation of the attributable risk: A loglinear approach. *Computational Statistics & Data Analysis.* 2012;56(12):4180-4189.
5. Hair JF, Tatham RL, Anderson RE, Black W. *Multivariate Data Analysis, 5th Ed.* Englewood Cliffs, N.J.: Prentice-Hall; 1998.
